# An Assessment of an Inpatient Robotic Nurse Assistant: A Mixed-Method Study

**DOI:** 10.1007/s10916-024-02117-4

**Published:** 2024-10-22

**Authors:** Yee Wei Lim, Shi Wei Tan, Cherylanne Yan Bing Tan, Dong Hee Michael Lee, Wen Ting Siow, Doreen Gek Noi Heng, Amartya Mukhopadhyay, Joo Cheng Lim, Sunil Sivadas, Ee Lin Kimberly Teo, Lawrence Khek Yu Ho, Jason Phua

**Affiliations:** 1 Medical Affairs — Research Innovation & Enterprise, Alexandra Hospital, National University Health System, Singapore, Singapore; 2 Department of Medicine, Yong Loo Lin School of Medicine, National University of Singapore, Singapore, Singapore; 3 NCS Pte Ltd, Singapore, Singapore

**Keywords:** Robotics, Nursing, Healthcare, Human–robot interaction

## Abstract

The worldwide nursing shortage has led to the exploration of using robotics to support care delivery and reduce nurses’ workload. In this observational, mixed-method study, we examined the implementation of a robotic nurse assistant (RNA) in a hospital ward to support vital signs measurements, medication, and item delivery. Human–robot interaction was assessed in four domains: usability, social acceptance, user experience, and its societal impact. Patients in a general medicine ward were recruited to participate in a one-time trial with the RNA and a post-trial 75-question survey. Patients’ interactions with the RNA were video recorded for analysis including patients’ behaviours, facial emotions, and visual attention. Focus group discussions with nurses elicited their perceptions of working with the RNA, areas for improvement, and scalability. Sixty-seven patients aged 21–79 participated in the trial. Eight in 10 patients reported positive interactions with the RNA. When the RNA did not perform to expectations, only 25% of patients attributed fault to the RNA. Video analysis showed patients at ease interacting with the RNA despite some technical problems. Nurses saw potential for the RNA taking over routine tasks. However, they were sceptical of real time savings and were concerned with the RNA’s ability to work well with older patients. Patients and nurses suggested greater interactivity between RNA and patients. Future studies should examine potential timesaving and whether time saved translated to nurses performing higher value clinical tasks. The utility of improved RNA’s social capability in a hospital setting should be explored as well.

**Supplementary Information:**

The online version contains supplementary material available at 10.1007/s10916-024-02117-4.

## Introduction

The World Health Organisation (WHO) projects a shortfall of 15 million healthcare workers [1] including a global shortage of 13 million nurses by 2030 [2]. Most countries will face challenges in employing and retaining healthcare workers. Nurses are the linchpin of hospital care [3]. Nursing work is physically and mentally laborious [4]. Studies have shown that fatigue impedes nurses’ ability to provide quality care [4] and is detrimental to their mental well-being [5]. Many countries are adopting alternatives to strengthen healthcare resilience such as autonomous technologies including robotics [6].

Robot-assisted healthcare potentially generates time and cost savings by automating routine tasks in day-to-day operations, allowing healthcare workers to focus on higher-level skills-based activities. Robotics systems have been implemented widely: they are part of pharmacy’s prescription systems [7], disinfection and goods transportation [8], surgical assistances, and social interaction with patients in nursing homes [9]. In addition, robots could reduce medical errors, improve diagnostic and treatment capabilities, and contribute to better quality of healthcare [8].

Despite increased utilization of robotics, user experience and perceptions remain relatively underexplored. Patients in a Los Angeles hospital reported modest acceptance of the social humanoid robot, Moxi, with patients worried that Moxi’s eyes may be a form of surveillance [10]. Several studies expressed concerns that robots may cause more harm than good if there is incongruence in users’ expectations and robot design. For example, HOBBIT, an anthropomorphic assistive robot, which only has assistive functions, caused displeasure among users who expected social functions due to HOBBIT’s human-like appearance [11, 12]. These examples highlight the need for further investigation of human–robot interactions (HRI) to better integrate robots into clinical settings [13].

**Fig. 1:**
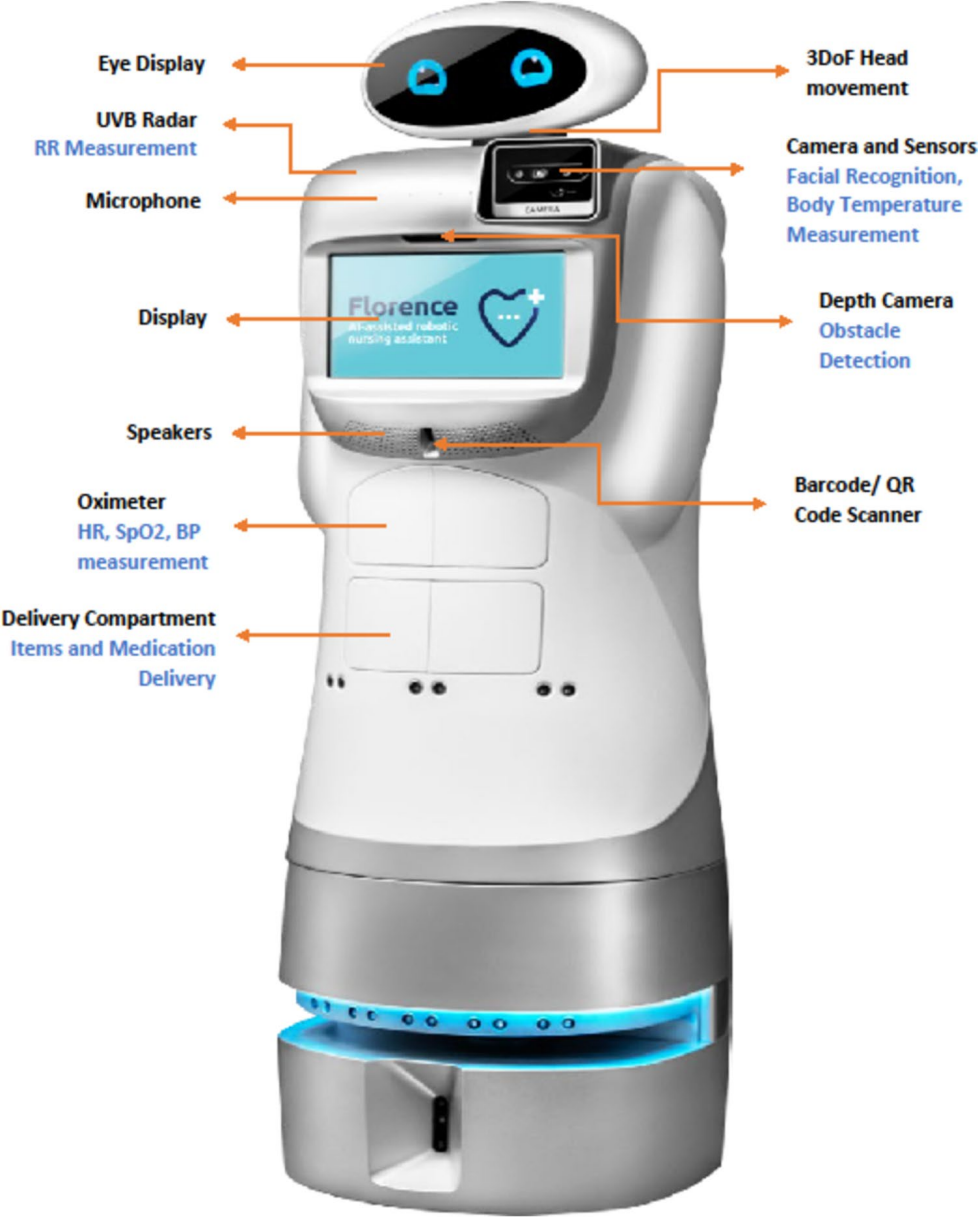
Image of Robotic Nurse Assistant, Florence, and its Functions

We examined Florence (Fig. 1), a humanoid robotic nurse assistant (RNA) that autonomously navigates within wards, locates, and identifies patients through facial recognition technologies. Florence interacted with patients through three tasks: (a) measuring patients vital signs (blood oxygen saturation, blood pressure, and body temperature) through an extending platform with a CNOGA oximeter, (b) delivering and dispensing medication pre-loaded in its front compartment, and (c) delivering small items such as refreshments in the same compartment. These tasks are depicted by video clips in the supplementary material section (Online Resource 1,2,3).

The objectives of this study were:

1. Assess the human–robot interaction in a dynamic inpatient setting;
2. Examine healthcare nurses’ assessment of the RNA’s functions and feasibility as a nurse assistant.

## Methods

The development and testing of Florence were described in an earlier conference proceeding [14]. We adopted a mixed-method approach to study HRI (Table 1). Three research assistants (RA) deployed Florence from the ward’s workstation to patients’ bedside to independently perform the three separate tasks and documented the patient-robot interactions. Data were collected through three modes: (a) video recording and observational notes of the patient-robot interactions, (b) post-trial patient experience survey, and (c) focus group discussions with nurses who worked with Florence. The study was conducted in a general medicine ward at Alexandra Hospital, an acute care hospital in Singapore.

**Table 1:** Overview of Data Collection Methods

S/N	Methods	Description	Purpose
1	Video Recording of the Trial with Florence	The video recording aimed to capture Florence in action for its three use cases	Investigate human–robot interaction based on patient’s conduct and physical reactions when interacting with Florence
		Trials were filmed with two action cameras:	
		• One was mounted near the screen to observe and recognise patients’ emotions through their facial expression	
		• The other was placed at the patient’s bedside to view the entire interaction including Florence’s movements in the ward, patient’s conduct, gestures and bodily movements in relation to the RNA	
2	Patient Experience Survey	A 30-min semi-structured interview for patients followed the trial with Florence	Investigation of human–robot interaction through patient’s viewpoint of their user experience
		The 75-question survey contained a mix of Likert scale and open-ended question. Questions were based on the USUS Framework. The survey aimed to glean insights from patients for the following:	
		• Ability of RNA to perform nursing task successfully	
		• Aesthetics of the RNA	
		• Perceptions and attitudes towards incorporating the RNA in the wards	
		• Social aspect of RNA interaction	
3	Focus group discussions with healthcare professionals	Focus group discussions with 11 nurses of different job grades (nurse managers, registered nurses and enrolled nurses) were conducted to evaluate the acceptance and attitudes towards usefulness of introducing Florence in the ward	Usability, feasibility, Scalability, Aesthetics, Safety and Improvements to be made on Florence from healthcare provider viewpoint
		A total of three focus groups were conducted across the study period, two in February 2022 and one in July 2022	

### Participants

Patients were eligible to participate if they were English-speaking, without physical or cognitive impairments, and able to provide consent. Patients were invited to a one-time trial with Florence and a post-trial survey. They were recruited through convenience sampling. The trial was conducted from October 2021 to July 2022.

### Data Collection and Analysis

HRI was analysed based on the Usability, Social acceptance, User experience, and Societal impact (USUS) Evaluation Framework [15]. The framework has been widely adopted by past HRI studies in healthcare [10].

Three RAs coded and analysed 390 videos of patient-robot interactions (verbal and nonverbal phenomena). Recordings covered four phases: 1. Florence entering the ward, 2. Navigation to the patient’s bedside, 3. Greeting the patient, and 4. Task performance. Interactions coded: verbal responses, behaviour/conduct, facial emotional recognition, and visual attention to the RNA’s actions. The first 60 videos were coded twice to ensure alignment of interpretation between coders. Examples of codes are provided in Online Resource 4. Interactions were quantified to indicate frequency of reactions.

Patients completed a 75-question survey (Table 2) after interacting with Florence. The development of the survey was guided by the USUS framework to examine anthropomorphism, task performance, and sociability. Survey data were analysed descriptively. Bivariate analyses were performed for selected subgroups. Open-ended questions’ answers were organized into themes. Categorical data were represented as percentages.

**Table 2:** Patient Experience Survey

Survey Questions
Use case Experience
Vital Signs Monitoring
1. Did the RNA explain its purpose to measure your vital signs to you clearly when it came into your ward?
2. How often did you feel that the RNA could understand your speech or gestures?
3. Did the RNA show you instruction videos to guide you on taking vital signs?
4. Were there enough instructions provided by the RNA video to guide you during the vital signs measurement?
5. How often were you able to have your vital signs measured successfully on your first try?
6. Why were you not successful on your first try?
7. Between the nurse and the RNA, who do you think is faster in terms of measuring your vital signs?
8. What are the problems you face when having the RNA measure your vital signs?
9. Do you think that the RNA is able to measure your vital signs independently without the need of a nurse? Why is that so?
Medication Delivery
10. Was it clear that the RNA was delivering medicine to you?
11. What problems did you face when the RNA assists you with medicine delivery?
12. Do you think that the RNA is able to help you deliver your medication independently without the need of a nurse? Why is that so?
Physical Attributes
13. I like the size of the RNA
14. I like the facial features of the RNA (e.g. face, ears, eyes, etc.)
15. The video screen on the RNA is big enough for me
16. The video quality on the RNA is clear enough for me
17. The RNA sounds like a machine
18. The volume in which the RNA speaks is just right for me to hear what it said
19. The speed in which the RNA speaks is just right for me to understand what it said
20. I prefer the RNA to speak in Singlish instead of standard English
21. I prefer the RNA to have a male voice instead of a female voice
22. I prefer that the RNA to look like a human
23. I like the overall design of the RNA
24. The RNA moves rigidly
25. The RNA seems prone to accidents when it moves around the ward
26. I like the RNA’s speed of movement
27. The RNA is noisy when it moves
Interacting with the RNA
28. In general, can you understand the RNA?
29. What improvements would you like to see for the design of the RNA?
30. The RNA greeted and interacted with me in a friendly manner
31. The RNA was courteous and respectful to me
32. The RNA makes me feel that technology is impressive
33. It would be convenient for me to have the RNA
34. I find the RNA easy to use
35. I think I can use the RNA without any help
36. The RNA is pleasant to interact with
37. I have confidence and trust in the RNA to do its tasks well
38. When interacting with the RNA, I felt like I was talking to a real person
39. If I should use the RNA, I would be afraid to break something
40. In your opinion, did the RNA work well together with other members of the staff caring for you?
41. Did your family members or friends interact with the RNA? If yes, what was their experience like? Did they like it?
42. During your stay here, did you observe the interactions between the RNA and other patients? If yes, what are your views about it?
Care Provision
43. Do you feel a difference between a nurse and RNA measuring your vital signs? Why or why not?
44. Do you feel a difference between a nurse and RNA delivering your medication? Why or why not?
45. I would like to continue having the RNA to be a part of the care team caring for you. Would you say you…?
46. What do you feel about having more RNAs in the wards to take care of you in the future? Why is that so?
47. Overall, do you feel that the RNA is safe to be used to assist you in your care? Why or why not?
Improvements/Suggestions
48. What do you like about the RNA?
49. What do you think could be improved?
50. What other features or functions would you like the RNA to have, other than the current ones you have experienced (item delivery, vital signs measurement and medicine delivery)?

Eleven nurses participated in focus group discussions (topic guide in Online Resource 5). Discussions were recorded and transcribed. Two RAs coded the discussions using a three-level thematic coding structure (themes, subthemes, and qualifying subthemes). Coded data were grouped according to the four components of the USUS framework (Table 3).

**Table 3:** Selected Quotes from Nurses during Focus Group Discussion

Themes	Quotes
Physical Attributes	“When I first saw the robot I was a bit disappointed, because it’s quite big but the compartments are a bit small for its size. Maybe if we can have some hooks or what, to hang clothes outside of the robot. It saves more space.” (Quote 1)
	“To me, I really like the—The eyes. the robotic nurse was able to express different kind of emotions like while waiting for the patient or while waiting for the face to be detected, then we can see what are the facial expressions. I think it can be quite entertaining as well, and I think it’s quite- to me I feel like entertaining! and it makes you feel a bit better somehow” (Quote 2)
Safety	“We purposely tested it out, whether it might cause an accident. I stood in front (of the RNA)… and then suddenly it braked and toppled a bit. I am scared that it will suddenly brake you see, because sometimes we couldn’t possibly know, especially coming out of cubicle. If it suddenly come in, because the nurses walk very fast, running to take things you know, then it suddenly brake. There is a potential safety issue.” (Quote 3)
	“When the robot takes vital signs for the patient, there’s really a wide gap between the robot and the patient itself. Imagine if the patient is a fall-risk patient, there’s still a risk that they might fall down while reaching over to the robot.” (Quote 4)
Feasibility	“Who will do all these task assignments? Will it be the nurse? One-by-one I will register the patient upon admission?…take photos?. Who will do all these things?” (Quote 5)
	“Maybe the robot can do everything on its own. I mean the robot is doing vital signs, then it will automatically go around the cubicle by itself, 1 2 3 4 5 beds. Once it is finished, the measurements will be recorded in the system, and then move on to another cubicle. Hopefully all this information can be automatically transmitted to the documentation system. It is able to flag things out. That would save a lot of time. This means that vital signs monitoring will not be part of the nurses’ work, it will just be done by the robot.” (Quote 6)
Usability	“The vital sign is excellent…It can help if someone can help us do that while we do other things, for example like when patient complains of chest pain, the robot can help do the vitals while we call the doctor.” (Quote 7)
	“It can save our time to do some other things, if we know someone (robot) is watching we can really focus on work. Otherwise, currently nurses… can’t go anywhere, they don’t dare to go for breaks just to make sure that the patients are safe.” (Quote 8)
	“For a person who can actually self-medicate, it may not be a problem. But 80% of the population needs assistance to take their medicine. The robot will just be there with the medicine, but we’ll still need to pour a glass of water and allow the patient to take the medication” (Quote 9)
	“For middle-age patients, there shouldn’t be a problem. But if you think about elderly patients…they will take some time to sit up and might have some difficulty sitting up. Unless you’re just taking the blood pressure and Florence come beside them and they can just lie down and insert their finger without sitting up. But I don’t know if it can be done.” (Quote 10)
	“I think generally medication- because medication process is very- it’s rather complicated. The thing is about making sure that the medication is identified- given to the patients correctly. The other thing it’s not just providing medicine, it’s a lot about education (of patients), that is the other component; The process of the nurse giving medication …it is not just the medicine is also to ensure that medicine is actually being taken, being fed and being given safelythen you’ve completed the process of administration. Not just the serving part of the processs. It’s the consumption that’s important.” (Quote 11)
Improvements	“If the robot can proactively ask: I am here to serve you, is there something that you want? On the screen there are 1,2,3,4,5’. The patient may choose ‘Okay, can you please get me some hot drinks’, then she presses number 5. If she needs to have one more blanket, then she presses the option for a blanket.” (Quote 12)
	“If it can make phone calls that would be helpful. Because in our ward, the older patients will make phone calls with the ward’s cordless phone. But they will usually ask us to call. If Florence can make video calls, like when they want to make video calls, send Florence and ask them to look at the camera. It’s like, ‘Oh I can see my family’..” (Quote 13)
	“I think it will be better if the robot can prepare medication itself and give to patient.” (Quote 14)

## Results

Sixty-seven patients (*n* = 37 males, *n* = 30 females) participated in the trial. Patients were aged 21–79, with 25 (37.3%) under 40, 22 (32.8%) aged 40–59, and 20 (29.9%) above 60 years old. Fifty-three patients completed the post-trial survey (Table 4). Bivariate analyses were performed to examine the relationship between gender, age, ethnicity, and educational level, and domains including social acceptance, usability, and user experience. No notable relationships were found. All bivariate results can be found in Online Resource 6.

**Table 4:** Participant characteristics for completed surveys

		*n* (%)
Age	≤ 40 years	22 (41.51%)
	40–59 years	19 (35.85%)
	≥ 60 years	12 (41.51%)
Gender	Males	30 (56.60%)
	Females	23 (43.40%)
Ethnicity	Chinese	34 (64.15%)
	Malay	12 (22.64%)
	Indian and Eurasian^1^	7 (13.21%)
Education level	Primary	6 (11.32%)
	Up to secondary	10 (18.87%)
	Post-secondary	37 (69.81%)

^1^ Demographic details collapsed into one category as there was only one Eurasian participant

### Video Analysis of HRI

#### User experience and social acceptance

Patients could socially interact effectively with the RNA. While no patients showed overt fear when they first encountered the RNA, six displayed observable hesitation—they had stiff gestures, unnatural postures, and was slow to respond to the RNA’s prompts. All patients paid attention to the RNA’s movements, 24% of patients smiling when it entered the cubicle. One in five patients acknowledged it with a “hi”, wave, or a nod when greeted. A handful of patients thanked the RNA for its service.

#### Usability

Patients’ use of the RNA was inconsistent. Some patients encountered difficulties with vital signs measurement. They were unaware of the voice-input function, 40% mistook the pain scale displayed on the screen for a touch-screen panel. One in two patients reported their pain score at least twice for the RNA to register it accurately. The oximeter could not fit the finger for 60% of patients due to improper finger placement and difficulties leaning forward to reach it. Patients showed visible discomfort when the distance between the RNA and their beds were wide causing them to strain their bodies. Patients interacted well for the medication and item delivery tasks.

### Patients Experience Survey

#### User experience and social acceptance

Eighty percent of patients had a positive experience interacting with the RNA and rated their comfort level 8.1 out of 10. Although 93% liked the RNA’s physical features and size, they were divided on whether it should be more human-like. Nineteen patients showed a fondness for the RNA’s facial expression, describing eye blinking motions “cute”. The RNA was deemed friendly and polite by 95% of patients. Patients preferred (53%) the RNA to have a female voice as it is more soothing and conforms to the conventional image of a nurse.

Patients exhibited high levels of social acceptance of the RNA; 83% were confident that the RNA can carry out its tasks well. The RNA was deemed safe to be utilised by 87% of patients as the tasks were considered “simple”, “programmed”, and “routine”, “requiring little human supervision”. Fifty percent trusted the RNA to perform tasks independently without intervention from nurses, while 89% trusted its use in the wards, and 72% believed it is not prone to accidents. Nine in 10 patients expressed interest for the RNA to continue caring for them.

#### Usability

Interactions with the RNA was reported to be easy by 94% of patients. They were comfortable using the RNA without assistance but believe that briefing is necessary before use. Patients were satisfied with the RNA’s performance of vital signs management. Seven in 10 patients understood the RNA’s gestures and instructions most of the time but there were concerns about dispensing wrong medications or misidentifying patient.

Although patients found the RNA simple to use and served its purpose, they also highlighted some limitations. One in five patients preferred faster navigational capabilities; 50% of patients reported long waiting time because of navigational errors and facial detection failures. One in 10 patients suggested the RNA could improve interaction by communicating in more languages. There was no agreement on whether nurses or the RNA was more efficient or provided superior care. Twenty-five patients reported little differences in medicine delivered by RNA and nurses. Fifteen patients shared that no differences in vital signs measurements was perceived. A few patients reported the RNA did not provide clear information about medicines. Patients reported that nurses took vital signs more quickly but required more equipment. In contrast, the RNA could do the task with a single integrated device. Patients compared RNA less favourably to nurses because it was less able to connect with them emotionally.

### Nurse Focus Group Discussions

#### Feasibility

The main theme emerging most strongly pertaining to feasibility was the impact of the RNA on their work processes. Nurses were optimistic about implementing the RNA in the ward and felt that automating tasks to ease their workload was an advantage. However, they were doubtful whether adoption of RNA will directly translate to increased productivity. Of the three use cases, nurses felt the vital signs monitoring task had the most potential in assisting nurses, but it took longer to perform the task. If the RNA could perform vital signs measurement on a fixed schedule, it could improve the ability to plan other tasks and potentially reduce workload, generating time-savings (Quote 6). Nurses further elaborated that confidence needs to be established for the glucometer’s accuracy. Some stated that reliability was crucial; unreliability would deter them from working with the RNA.

#### Usability

Two themes emerged from the objective of usability. The first concerns the operating of the RNA. Nurses expressed scepticism over the process of preparation of the RNA for medication and item delivery tasks. The need to retrieve an item from the ward, load it into RNA’s compartment, and activate the RNA through its dashboard, was cumbersome. They reported it was more straightforward to bring the items directly to the patient. There were concerns that frail patients might drop or spill the medication while retrieving the item from the RNA. The RNA only instructs patients to consume their medication and has no mechanism to ascertain medication consumption, which was regarded as an area for improvement, such as including a verification step that confirmed patients have taken the medications (Quote 11). Integration of the RNA’s programming into their current workflow was also a concern, such as installation of the RNA’s operating system in the wards, as well as integration of data with the electronic medical record system.

Usability of the RNA is also dependent on the inpatient environment. Nurses opined that the RNA was not suitable for several patient types, in particular, those with limited mobility, cognitive impairments, or limited English comprehension. They were concerned that high human traffic during visiting hours may disrupt the robot’s in-built navigation routes. A nurse manager suggested that the RNA only worked within a single cubicle to minimise navigational challenges. Nurses could assign appropriate patients to be cared for by the RNA in this cubicle (i.e. younger, English-speaking, ambulant).

Nurses suggested the RNA could have additional capacity to carry and transfer larger items like blankets. Social companionship could be further developed through better communication capabilities (Quote 12), such as engaging patients in two-way conversations.

## Discussion

Our study found the RNA was generally perceived in a positive light. Patients engaged with the RNA to complete functions and found its anthropomorphic features pleasing. Nurses saw the potential of the RNA freeing up their time so they could provide more direct patient care. However, we found a few technical challenges; the RNA did not meet expectations in performing vital signs monitoring, medication delivery did not guarantee reliability, and RNA’s movement could be faster. Patients and nurses suggested to include social interaction function to enhance RNA’s role in the ward. The RNA’s ability to save time would require further investigation.

### Social Acceptance

We found patients had positive interactions with the RNA. Patients smiled at the RNA and thanked it for its service as they would to a human being. RNA’s female voice and human-like torso might have conjured an image of a nurse, which allowed patients to relate with the RNA more comfortably [16, 17]. We suspect the ubiquity of robots in daily lives in Singapore, such as cleaning robots in malls, and the frequent depiction of robots in popular culture, also contributed to a sense of familiarity to its existence and a high level of acceptance [18].

### User Experience and Usability

Patients were generally satisfied with the RNA’s performance across the three tasks. They indicated good user experiences despite experiencing errors and having to play a more active role. They were most comfortable with the simpler function of medication and item delivery. For more elaborate processes of vital signs measurement, the RNA’s instructions were not as well understood and more than half of the patients made multiple attempts using the oximeter and voice-input pain score. Even after experiencing these challenges, only a quarter attributed it to insufficient instructions provided. Patients attributed the difficulties to “patient-as-user-issues”, “lack of familiarity”, and perceived the RNA to function well in their recount of the trials. Patients’ willingness to accommodate the RNA could be their lowered expectation that the RNA was not a perfect replacement for nurses [19]. Imperfections in social robots have shown to increase likability as they appear less distant [20].

Patients desired more social interaction capabilities beyond basic greetings. They suggested more two-way conversations, to better replicate how a nurse would communicate with patients. This feedback highlights the multifaceted nature of nursing care—going beyond clinical tasks and the importance of human interaction in the nurse-patient relationship, which is an area of ongoing and future development [21–23].

### Long-Term Feasibility

Patient surveys and nurse focus group discussions revealed the concept of time efficiency is not straightforward and has different implications for different stakeholders. Patients experienced long wait times because the RNA is programmed to move slowly and sometimes required multiple attempts at their tasks. This could disrupt patient’s rest and recovery. For nurses, it was perceived to be acceptable that the RNA took longer to perform, in particular lower value tasks, such as item delivery. However, for higher value tasks, such as medication delivery, the trade-off between speed and reliability would need to be considered more carefully as it has serious implications.

Time efficiency is further complicated by the value which task substitution adds. While studies in some sectors viewed robotics more favourably if it improved task efficiency, accuracy, consistency, and speed [24, 25], other studies perceived robots to be valuable when substituting humans in time-consuming and repetitive tasks even if it took longer to perform [26]. At present, the RNA is unable to independently perform as it relies on nurses to allocate it tasks. If vital signs measurement could be programmed in advance and performed on a fixed schedule, it would improve RNA’s independence. Another approach would be to develop a more intuitive learning system that could facilitate better understanding of patients’ needs and respond more effectively (such as delivering a glass of water). With increase in RNA’s independence, the time freed up could provide nurses take on tasks better performed by humans, such as patient education and emotional support or more time for rest [24, 27]. Our study suggests there is a need to examine whether time efficiency or task substitution adds value to a nurse’s work life. One approach would be to perform a time and motion study of robots carrying out nurse-related tasks to ascertain potential time-savings generated by the RNA and at the same time examine how the time saved is used by nurses [28, 29]. For example, in a study of a pharmacy dispensary robot, researchers found an increase in work productivity and reduced staff movement in the dispensary—this could be explored for the RNA in a ward [30].

### Feasibility for Diverse Patient Profiles

Nurses expressed concerns that older adults, who are the main group of patients in a general medical ward, might have difficulties interacting with the RNA. More work is required to examine the role of patient’s physical limitations, cognitive impairment, and cultural factors that could pose as barriers for RNA’s adoption. Customization and better matching of robot to local human context would be required as those who less tech savvy might find it harder to interact with the RNA [31–34].

### Limitations

This study has several limitations. First, a bias could have been introduced because patients were briefed prior to the trial and agreed to spend a considerable time to test the RNA’s functions. Patients who voluntarily agreed to the trial might have had higher technological acceptance, thus influencing their experience with the RNA. The participant pool was limited to English-speaking patients, patients without mobility constraints and cognizant to use the device. This reduced other scenarios for examining human–robot interactions. Second, each patient only tested the RNA in a one-time supervised trial; if we repeated the trial several times, we might have found additional insights and problems arising from implementing the RNA in an acute ward setting. Third, this study assessed one robot; other RNA prototypes deployed elsewhere might have performed differently.

### Future Research Work

Our study findings suggest a few areas for further exploration: enhancing social interactivity, improving medication administration, and studying the RNA’s ability to ease nursing workload.

Improving social interactivity could expand the scope and function of the RNA. Future research could examine two-way interactions between the RNA and users, such as reactions to patients’ responses [30]. Information obtained during interactions could be conveyed to nurses to alert them to patients who require attention or learn more about their patients, improving the patient-provider relationship [35, 36].

Nurses are concerned that non-compliant patients may not consume medicines delivered by the RNA. The feasibility of incorporating sensors [36, 37], action-validation functions, and persuasive abilities [38–40] into the RNA’s ecosystem to verify medication consumption and encourage medical adherence may be examined.

A detailed study is needed to assess whether robots can substantially ease nurses’ workload [41]. We propose a time and motion study of robots carrying out nurse-related tasks to ascertain time-savings generated by the RNA and simultaneously document how nurses use the time saved by the RNA [29].

## Conclusion

RNA was perceived positively by patients and nurses but there were concerns about some of its technical functions and how it could be integrated into nursing workflow. Future studies should explore RNA’s social interactivity and its ability to ease nurses’ workload.

## Supplementary Information

Below is the link to the electronic supplementary material.

Supplementary file1 (MP4 5989 KB)

Supplementary file2 (MP4 2493 KB)

Supplementary file3 (MP4 3490 KB)

Supplementary file4 (DOCX 29 KB)

Supplementary file5 (DOCX 14 KB)

Supplementary file6 (DOCX 42 KB)

Supplementary file7 (XLSX 74 KB)

Supplementary file8 (DOCX 124 KB)

Supplementary file9 (DOCX 44 KB)

## Data Availability

No datasets were generated or analysed during the current study.

## Abbreviations

RNA: Robotic nurse assistant
HRI: Human–robot interaction
