# Supplementary material for: An Assessment of an Inpatient Robotic Nurse Assistant: A Mixed-Method Study
Source: J Med Syst. 2024 Oct 22;48(1):99. doi: 10.1007/s10916-024-02117-4 (PMC11496348; doi:10.1007/s10916-024-02117-4)
Supplement: Supplementary file 4 — Supplementary file4 (DOCX 29 KB) [file 10916_2024_2117_MOESM4_ESM.docx]

Appendix B

Examples of video coding of patient-robot interaction

| **Video number** | **Step** | **RNA Tasks** | **Timestamp**  **(mm:ss)** | **Description of Human-Robot Interaction (HRI)** | | | | **Coder** |
| --- | --- | --- | --- | --- | --- | --- | --- | --- |
|  |  |  |  | **Verbal** | **Behavioral/Conduct** | **Facial Emotional Recognition (FER)** | **Visual (Attention)** |  |
| PT002 VSM | 01 | Approaches patient and maneuvers to face patient |  |  |  | Neutral expression. | Patient kept his gaze on the RNA. | CT |
|  |  |  |  |  | PT was sitting upright in bed, with his legs outstretched and crossed | Neutral/relaxed expression | Maintained eye contact with RNA | YW |
| PT003 VSM | 01 | Approaches patient and maneuvers to face patient |  |  | Sat upright on his bed. | Had a neutral expression. | Patient kept his gaze on the RNA. Gazed at the RNA’s camera as it came out to detect patient. | CT |
|  |  |  |  |  | Sat upright at the side of the bed with his wrist tag held in his right hand | Brows were furrowed, seemingly concentrating/ evaluating the RNA | Gaze fixed on RNA | YW |
| PT007 VSM | 08 | Compartment opens, and request patient to insert finger into oximeter |  |  | Raised his hand to put into the platform before RNA finished her verbal instructions, but seemed unsure if he was doing it correctly. Retrieved back and scratched his head, before putting his finger into the oximeter. Hand was in a stiff position; patient did not rest his wrist on the platform. | Gaze shifted to look and read instructions on the RNA screen, then glanced at the opened compartment and the platform coming out.    Patient’s gaze fixed on the RNA screen throughout the measurement. |  | CT |
|  |  |  |  |  | Stretched out his hand to place in oximeter before the instructions were complete, but retrieved it at the last moment and scratched his head, seemingly unsure as to if there were further instructions or as to where to position his hand.    He then placed his finger in the oximeter, but his forearm was not rested on the platform. | Looked slightly confused | Eye contact shifted between compartment and video screen. | YW |
| PT053 VSM | 08 | Compartment opens, and request patient to insert finger into oximeter |  |  | Patient looked carefully at the RNA’s screen and was hesitant about her next move as she pressed her lips together in concentration.    Patient then hesistantly reached forward to insert her finger into the oximeter. | Patient had a careful look on her face. | Patient was focused on the oximeter but looked towards the screen for any prompts as she waited. | SW |
| PT012 ID | 01 | Approaches patient and maneuvers to face patient |  |  | Patient remained still on her bed, with her two hands in her lap as she waited for the RNA to approach her. | Patient had a slight smile on her face. | Patient scanned the length of the RNA’s body before glancing up at her head and down to her screen and compartment. | SW |
| PT053 ID | 01 | Approaches patient and maneuvers to face patient |  |  | Patient was sat upright in her bed that was propped up. She was gesturing animatedly to her friend that she can stand closer to the RNA.    When the RNA finally managed to locate the patient, she made a lengthy comment about it to the RA. | Patient had a neutral expression on her face.        Patient looked confused but amused at the RNA’s error. | Patient was focused on her conversation with her friend, but paid some attention to the RNA. | SW |
| PT026 MD | 06 | Compartment opens for patient to retrieve medication. |  |  | Patient stretched out her arm and shifted her body closer to the RNA to retrieve medication (seemingly a stretch, and took a few seconds before successfully capturing the barcode) | Neutral expression | Gaze fixed on the compartment, and then to the medication upon retrieval | CT |
| PT053 MD | 06 | Compartment opens for patient to retrieve medication. |  | “Haha so cute one”      “Haha” | Patient looked straight at the compartment and laughed.      Patient laughed again when RNA prompts her to consume the medication. | Patient looked amused. | Patient glanced between the compartment and the RNA’s screen. | SW |
