# Supplementary material for: An Assessment of an Inpatient Robotic Nurse Assistant: A Mixed-Method Study
Source: J Med Syst. 2024 Oct 22;48(1):99. doi: 10.1007/s10916-024-02117-4 (PMC11496348; doi:10.1007/s10916-024-02117-4)
Supplement: Supplementary file 5 — Supplementary file5 (DOCX 14 KB) [file 10916_2024_2117_MOESM5_ESM.docx]

Appendix C

Staff focus group discussion topic guide

| 1. What were your expectations of the RNA when it was first introduced? How did you feel about it? 2. Describe your experience of working with the RNA. Were your expectations of the RNA met? Has your perception of the RNA changed as a result of working with it? How so? 3. Has the use of RNA changed your workload, in terms of burden or space? Did you have trouble using the RNA? How so? 4. Which aspects of the RNA have worked well? 5. Which aspects of the RNA did not work well? 6. Do you think the RNA has been received well by you or your colleagues? Has this changed over time? What did they share with you about their experiences with the RNA? 7. Do you think the RNA has been received well by the patients? Has this changed over time? 8. Do you consider the RNA to be social? Why or why not? 9. How did you feel when the RNA approached you in the hallways? 10. What have been the challenges of implementing the RNA in the wards? 11. What improvements would you make to the RNA? 12. Do you think the RNA should continue to be used? Would you want to continue using the RNA? How do you think life would change if the RNA was integrated into the hospital permanently? |
| --- |
