# Supplementary material for: An Assessment of an Inpatient Robotic Nurse Assistant: A Mixed-Method Study
Source: J Med Syst. 2024 Oct 22;48(1):99. doi: 10.1007/s10916-024-02117-4 (PMC11496348; doi:10.1007/s10916-024-02117-4)
Supplement: Supplementary file 6 — Supplementary file6 (DOCX 42 KB) [file 10916_2024_2117_MOESM6_ESM.docx]

Online Resource 6: Selected bivariate analyses of relationship between demographic characteristics and questions about acceptability, usability, and user experience

Question: Do you feel a difference between a nurse and RNA measuring your vital signs?

|  | No | Yes | Pearson chi-square |
| --- | --- | --- | --- |
| **Age** |  |  |  |
| < 50 years | 8 | 22 | χ^2^ (1) = 0.17  *p* = .68 |
| ≥ 50 years | 5 | 18 |  |
|  |  |  |  |
| **Gender** |  |  |  |
| Female | 8 | 15 | χ^2^ (1) = 2.31  *p* = .13 |
| Male | 5 | 25 |  |
|  |  |  |  |
| **Educational level** |  |  |  |
| Up to secondary | 6 | 10 | χ^2^ (1) = 2.08  *p* = .15 |
| Post-secondary | 7 | 30 |  |
|  |  |  |  |
| **Ethnicity** |  |  |  |
| Chinese | 5 | 29 | χ^2^ (2) = 6.04*  *p* = .05 |
| Malay | 6 | 6 |  |
| Indian and Eurasian | 2 | 5 |  |

^*^ *p* ≤ .05

Question: I would like to continue having the RNA to be a part of the care team caring for you.

|  | Agree | Disagree | Pearson chi-square |
| --- | --- | --- | --- |
| **Age** |  |  |  |
| < 50 years | 28 | 0 | χ^2^ (1) = 2.53  *p* = .11 |
| ≥ 50 years | 21 | 2 |  |
|  |  |  |  |
| **Gender** |  |  |  |
| Female | 22 | 1 | χ^2^ (1) = 0.02  *p* = .89 |
| Male | 27 | 1 |  |
|  |  |  |  |
| **Educational level** |  |  |  |
| Up to secondary | 14 | 1 | χ^2^ (1) = 0.43  *p* = .51 |
| Post-secondary | 35 | 1 |  |
|  |  |  |  |
| **Ethnicity** |  |  |  |
| Chinese | 31 | 2 | χ^2^ (2) = 1.14  *p* = .57 |
| Malay | 11 | 0 |  |
| Indian and Eurasian | 7 | 0 |  |

Question: I like the size of the RNA.

|  | Agree | Disagree | Pearson chi-square |
| --- | --- | --- | --- |
| **Age** |  |  |  |
| < 50 years | 28 | 1 | χ^2^ (1) = 2.87  *p* = .09 |
| ≥ 50 years | 19 | 4 |  |
|  |  |  |  |
| **Gender** |  |  |  |
| Female | 20 | 3 | χ^2^ (1) = 0.56  *p* = .46 |
| Male | 27 | 2 |  |
|  |  |  |  |
| **Educational level** |  |  |  |
| Up to secondary | 15 | 1 | χ^2^ (1) = 0.30  *p* = .58 |
| Post-secondary | 32 | 4 |  |
|  |  |  |  |
| **Ethnicity** |  |  |  |
| Chinese | 29 | 4 | χ^2^ (2) = 1.01  *p* = .61 |
| Malay | 11 | 1 |  |
| Indian and Eurasian | 7 | 0 |  |

Question: I like the facial features of the RNA (e.g. face, ears, eyes etc).

|  | Agree | Disagree | Pearson chi-square |
| --- | --- | --- | --- |
| **Age** |  |  |  |
| < 50 years | 29 | 1 | χ^2^ (1) = 0.70  *p* = .40 |
| ≥ 50 years | 21 | 2 |  |
|  |  |  |  |
| **Gender** |  |  |  |
| Female | 22 | 1 | χ^2^ (1) = 0.56  *p* = .46 |
| Male | 28 | 2 |  |
|  |  |  |  |
| **Educational level** |  |  |  |
| Up to secondary | 15 | 1 | χ^2^ (1) = 0.01  *p* = .90 |
| Post-secondary | 35 | 2 |  |
|  |  |  |  |
| **Ethnicity** |  |  |  |
| Chinese | 31 | 3 | χ^2^ (2) = 1.78  *p* = .41 |
| Malay | 12 | 0 |  |
| Indian and Eurasian | 7 | 0 |  |

Question: Overall, do you feel that the RNA is safe to be used to assist in your care?

|  | No | Yes | Pearson chi-square |
| --- | --- | --- | --- |
| **Age** |  |  |  |
| < 50 years | 3 | 26 | χ^2^ (1) = 0.04  *p* = .84 |
| ≥ 50 years | 2 | 21 |  |
|  |  |  |  |
| **Gender** |  |  |  |
| Female | 1 | 22 | χ^2^ (1) = 1.32  *p* = .25 |
| Male | 4 | 25 |  |
|  |  |  |  |
| **Educational level** |  |  |  |
| Up to secondary | 2 | 14 | χ^2^ (1) = 0.22  *p* = .64 |
| Post-secondary | 3 | 33 |  |
|  |  |  |  |
| **Ethnicity** |  |  |  |
| Chinese | 4 | 30 | χ^2^ (2) = 0.84  *p* = .66 |
| Malay | 1 | 11 |  |
| Indian and Eurasian | 0 | 6 |  |

Question: The RNA greeted and interacted with me in a friendly manner.

|  | Agree | Disagree | Pearson chi-square |
| --- | --- | --- | --- |
| **Age** |  |  |  |
| < 50 years | 17 | 12 | χ^2^ (1) = 0.22  *p* = .64 |
| ≥ 50 years | 12 | 11 |  |
|  |  |  |  |
| **Gender** |  |  |  |
| Female | 11 | 12 | χ^2^ (1) = 1.05  *p* = .30 |
| Male | 18 | 11 |  |
|  |  |  |  |
| **Educational level** |  |  |  |
| Up to secondary | 12 | 4 | χ^2^ (1) = 3.46  *p* = .06 |
| Post-secondary | 17 | 19 |  |
|  |  |  |  |
| **Ethnicity** |  |  |  |
| Chinese | 19 | 14 | χ^2^ (2) = 2.72  *p* = .26 |
| Malay | 8 | 4 |  |
| Indian and Eurasian | 2 | 5 |  |

Question: The RNA makes me feel that technology is impressive.

|  | Agree | Disagree | Pearson chi-square |
| --- | --- | --- | --- |
| **Age** |  |  |  |
| < 50 years | 22 | 8 | χ^2^ (1) = 1.47  *p* = .23 |
| ≥ 50 years | 20 | 3 |  |
|  |  |  |  |
| **Gender** |  |  |  |
| Female | 19 | 4 | χ^2^ (1) = 0.28  *p* = .60 |
| Male | 23 | 7 |  |
|  |  |  |  |
| **Educational level** |  |  |  |
| Up to secondary | 14 | 2 | χ^2^ (1) = 0.95  *p* = .33 |
| Post-secondary | 28 | 9 |  |
|  |  |  |  |
| **Ethnicity** |  |  |  |
| Chinese | 25 | 9 | χ^2^ (2) = 1.98  *p* = .37 |
| Malay | 11 | 1 |  |
| Indian and Eurasian | 6 | 1 |  |

Question: It would be convenient for me to have the RNA.

|  | Agree | Disagree | Pearson chi-square |
| --- | --- | --- | --- |
| **Age** |  |  |  |
| < 50 years | 23 | 4 | χ^2^ (1) = 0.03  *p* = .86 |
| ≥ 50 years | 20 | 3 |  |
|  |  |  |  |
| **Gender** |  |  |  |
| Female | 18 | 5 | χ^2^ (1) = 2.12  *p* = .15 |
| Male | 25 | 2 |  |
|  |  |  |  |
| **Educational level** |  |  |  |
| Up to secondary | 14 | 2 | χ^2^ (1) = 0.04  *p* = .83 |
| Post-secondary | 29 | 5 |  |
|  |  |  |  |
| **Ethnicity** |  |  |  |
| Chinese | 26 | 5 | χ^2^ (2) = 0.44  *p* = .80 |
| Malay | 11 | 1 |  |
| Indian and Eurasian | 6 | 1 |  |

Question: I find the RNA easy to use.

|  | Agree | Disagree | Pearson chi-square |
| --- | --- | --- | --- |
| **Age** |  |  |  |
| < 50 years | 28 | 2 | χ^2^ (1) = 0.13  *p* = .72 |
| ≥ 50 years | 22 | 1 |  |
|  |  |  |  |
| **Gender** |  |  |  |
| Female | 20 | 3 | χ^2^ (1) = 4.15  *p* = .04* |
| Male | 30 | 0 |  |
|  |  |  |  |
| **Educational level** |  |  |  |
| Up to secondary | 16 | 0 | χ^2^ (1) = 1.38  *p* = .24 |
| Post-secondary | 34 | 3 |  |
|  |  |  |  |
| **Ethnicity** |  |  |  |
| Chinese | 32 | 2 | χ^2^ (2) = 0.58  *p* = .75 |
| Malay | 11 | 1 |  |
| Indian and Eurasian | 7 | 0 |  |

**p* < .05

Question: Between the nurse and the RNA, who do you think is faster in terms of measuring your vital signs?

|  | Nurse | RNA | No difference | Pearson chi-square |
| --- | --- | --- | --- | --- |
| **Age** |  |  |  |  |
| < 50 years | 16 | 8 | 6 | χ^2^ (2) = 2.43  *p* = .30 |
| ≥ 50 years | 10 | 10 | 8 |  |
|  |  |  |  |  |
| **Gender** |  |  |  |  |
| Female | 12 | 6 | 5 | χ^2^ (2) = 1.99  *p* = .37 |
| Male | 14 | 12 | 8 |  |
|  |  |  |  |  |
| **Educational level** |  |  |  |  |
| Up to secondary | 8 | 7 | 1 | χ^2^ (2) = 1.81  *p* = .40 |
| Post-secondary | 18 | 11 | 7 |  |
|  |  |  |  |  |
| **Ethnicity** |  |  |  |  |
| Chinese | 18 | 10 | 5 | χ^2^ (4) = 3.38  *p* = .50 |
| Malay | 5 | 4 | 3 |  |
| Indian and Eurasian | 3 | 4 | 0 |  |

Question: Did the RNA explain its purpose to measure your vital signs to you clearly when it came into the ward?

|  | No | Yes | Pearson chi-square |
| --- | --- | --- | --- |
| **Age** |  |  |  |
| < 50 years | 2 | 27 | χ^2^ (1) = 3.63  *p* = .06 |
| ≥ 50 years | 6 | 17 |  |
|  |  |  |  |
| **Gender** |  |  |  |
| Female | 4 | 19 | χ^2^ (1) = 0.13  *p* = .72 |
| Male | 4 | 25 |  |
|  |  |  |  |
| **Educational level** |  |  |  |
| Up to secondary | 3 | 13 | χ^2^ (1) = 0.20  *p* = .65 |
| Post-secondary | 5 | 31 |  |
|  |  |  |  |
| **Ethnicity** |  |  |  |
| Chinese | 7 | 26 | χ^2^ (2) = 3.04  *p* = .22 |
| Malay | 1 | 6 |  |
| Indian and Eurasian | 0 | 12 |  |
