# Supplementary material for: An Assessment of an Inpatient Robotic Nurse Assistant: A Mixed-Method Study
Source: J Med Syst. 2024 Oct 22;48(1):99. doi: 10.1007/s10916-024-02117-4 (PMC11496348; doi:10.1007/s10916-024-02117-4)
Supplement: Supplementary file 8 — Supplementary file8 (DOCX 124 KB) [file 10916_2024_2117_MOESM8_ESM.docx]

**Interview with IDI1**

**IDI ID number:** __IDI1__

**Date of IDI:** 7^th^ February 2022

**Moderator Number:** __1___

**Notetaker Number:** __1__

**Number of IDI participants:** __5___

**Type of IDI Participants:** ____Nurses__

**Time IDI started:** ____2.30pm _____

**Time IDI ended:** _____3.30pm____

**Annotated notes:**

M: Moderator

IDI1_1: Informant 1

IDI1_2: Informant 2

IDI1_3: Informant 3

IDI1_4: Informant 4

IDI1_5: Informant 5

“XXX”: Direct quotes by the informant or the interviewer

{XXX}: My best guess/paraphrase of what he said

~ : Missing words or statements

[XXX]: My comments and description, gestures

… : Pauses

Start

M: “So erm, I assume that all of you have in some form or another interacted with the RNA right? [Participants agree in the background, “Yes” and “Um”.] Maybe I will start with IDI11, how- how have you been working with the RNA?”

IDI11: “Erm, so far okay because erm, it can help- reduce our workload la, by helping us to deliver the erm **medication** to the patient, ya. [M: “Okay.”] Um.”

M: “IDI12, have you handled the RNA as well?”

IDI12: “Oh yes, that day I- I did participate to do it. Erm, ya it can reduce the workload, however there is another point of- a bit of interaction ah. Because it’s a robot, when the nurses some are busy, so it’s double working.”

M: “Right, IDI13 how did the- what did you do with the RNA?”

IDI13: “Actually I didn’t really physically like key in the things, erm – I just watched, like just observed what the robot can do.”

M: “Um, I see. IDI14 leh?”

IDI14: “I think we erm… for me is looking through the- the process from the time when we initially set up in ward er, ward 12 we were-. That was the initial phase la. Then subsequently it was actually more in ward 4, so the process from the time whereby the robot moved from one- erm the main station and then to complete the whole process of it la. [M: “I see.”] Ya, about the three tasks.”

M: “IDI15, did you co-operate the RNA ah?”

IDI15: “I was involved since the customization of how the robot will look like, [M: “Ah…”] how the eyes will blink all these. Ya, so I- I can say that I see how the robot progress ah. But, for the three tasks that the robot can do, what I like the most is the vital signs monitoring. Because the medication is just a last-mile, delivering medication to patient [M: “Correct.”] I think it will be better if the robot can prepare **medication** itself and give to patient la.”

M: “We’ll dive into that deeper.”

IDI15: “Yes, and the other thing is the delivery of items ah, there’s such a, what it is ah- limitation on what are the- what are the **items** that we can put inside to the robot, because the compartment is quite small la. So with the three tasks, I think the vital signs better.”

M: “Of the 5 of you, did anyone actually operated the RNA from the resting dock to the patient, or is it all mostly done by [Note taker] is it?”

IDI15 and IDI13: “Ya.”

M: “So the nursing- our nursing colleagues didn’t have to-.”

Note taker: “We did let them try the- using the dashboard.”

M: “Dashboard- did anyone try the dashboard?”

IDI15: “I think they taught us la, they showed us how to do it, but we never really hands-on.”

M: “Oh, okay. We will talk about the dashboard shortly, erm maybe we’ll start with the outside-in. Or maybe we should start with function ah. So the three use case right, vital sign, medication delivery and erm, delivering items. And erm, IDI15 already mentioned that the last two wasn’t as useful. I’d like to take the vital signs first, what do you all think ah? The vital signs and how the RNA has performed doing it ah?”

IDI12: “Oh, okay. So what I see right, the robot will go there right, it need to capture the patient, scan first. So the patient able to move the scan too, provided this patient is correct la. So the- he will be looking at to face the patient **scanning** part, so in the first part, patient is- need to remove whatever you know, need to capture, so it can be erm… improve your technology that you know like CCTV like that whoever capture la. Ya, so he’s standing a bit far, RNA. The- he need to capture where is the patient. The patient have to move and show the hand. So it’s a bit far away, so um-.”

M: “I see- have to extend his hand, move the face. [IDI12: “Correct.”] So that it faces the robot’s **camera** right?”

IDI12: “Yes, correct. So it can be improved, how you are- you know that kind of how the camera will be capture the patient. Instead of patient have to follow to move la. [M: “Right, I see.”] So for the patient’s safety la. That’s one la huh.”

M: “I see, what do the other thinks? Do the others think- share the same thoughts?”

IDI15: “I do agree with her. [IDI14: “Um.”] Because we go and observe how the – the robot take the **vital** **signs** for the patient right, there’s really a **wide gap** between the robot and the patient itself. So imagine if the patient is a fall-risk patient, ya – there’s still a risk that they fall down while reaching to the- to the robot.”

M: “I see, I see. Other thoughts about this particular issue?”

IDI14: “I think same thing, is reaching – reaching is one point la, erm and then because we also know that the selection of patients is actually quite limited right, that we view once patients that are actually quite well into- no mal- no cognitive impairments and etcetera in this pilot phase, so the other thing is let’s say one of the thoughts is that let’s say if those patient who actually may not be able to really **follow instructions**… ya, then that may be a limitations la, let’s say if we move forward. And then the other thing is that we also know the capacity now is just doing your pulse rate, you know your saturation. Ya, so the other part is let’s say you want to enhance it, then whether your other things like your blood pressure monitoring and all that would be able to embed into the whole system, ya.”

M: “If- if I were to take up the points point by point, and I’d like to hear from IDI13, [IDI13: “Ya, okay so-.”] the- the-. Ya.”

IDI13: “So they’ve erm, said like most of my thoughts, but I actually just want to add maybe **infection** **control** la, because if the robot is going from one patient to another, is the nurse still have to go and wipe it la. So this-.”

M: “Right, right. I’d like to dive a little deeper the three points, the point of the patient’s distance from the RNA, am I correct to say right, paraphrase, it would be good if the RNA was able to **move closer** to the patient to the bedside?”

IDI1_participants: “Ya, yes.”

M: “Right now is a bit too far-. [IDI12: “Too far and ya-.”] And the- is it because of the size of the RNA? [IDI12 AND IDI13: “No.”] Or because there are too many things in front of the patient’s bedside? [IDI1_participants: “No.”]”

IDI13: “It’s just an empty **gap**, so-.”

IDI12: “Ya, um-.”

M: “So- is it because the wheels are too big-. [IDI12: “No.”] that the RNA can’t move closer to the bedside ah?”

IDI12: “No actually the RNA can move to nearer to look for the patient, because it was standing at the edge of the bed, so the patient head is other side. So it was standing a bit far, he looking where is the patient. Ya, ya. [M: “I see, I see.”] Actually it can move to nearer and capture the patient.”

M: “And the **facial recognition** part is erm, inconvenient is that correct?”

IDI15: “Because the patient need to sit upright. Ya, then the certain angle the RNA cannot capture.”

[IDI1_participants agree in the background.]

M: “Is it- how can we overcome, is it- would be, that the RNA already have inherent images of the patient before, so that they don’t have to have that extra step? What would be a solution to that ah?”

IDI12: “I mean you can capture more, or turn or move more, more cooperate on the movement side la. Because it will be focus on one side only. Ya, so if your RNA the head right can able to capture the patient where is it, scan to see the patient and he can move and do lor. But I have a feeling like-. (laughs)”

M: “[Note taker], if I am not wrong right, the photo taking is to make sure that there is validation of the name and the face of the patient right? [Note taker: “Um.”] So that’s a step that cannot be avoided right?”

Note taker: “Ya. So there’s like a two-step of identification, so like there will be a wrist tag, and there will be like a facial detection. Ya.”

M: “And… reading between the lines, you all think that is a bit of a hassle, is it correct? This two-step process.”

IDI14: “I think number one is that we know, because we observe them during the **facial scanning** right, you have to scan different different- face different angles, that’s the- even before the implementation process itself la, so erm prior to that is the five- five area scanning of the face itself, of the client. That’s one already, then number two is that let’s say if you want to- for the robot to recognize the client usually you have to really face it to the camera, then you will be able to capture the identity of the client la.”

M: “Hypothetically, if scanning just the wristband, is that enough ah? For verification, what do you think ah?”

IDI14: “Erm, generally I think this is what we are doing.”

IDI15: “I don’t know how the robot is being programmed, but in the ground, we actually identify the patient to the wrist tag, but of course we still ask la. Ask them to tell us their name and IC [M: “IC number.”] la. Ya.”

M: “Right, so there’s still a double verification process la.”

IDI12: “Yes, actually robot can say what is your name and then what is you IC number. That is because with the nurses we ask the patient, those who are cognitively good la. So erm, okay so Mr so-and-so, what is your full name and your IC, that is sometimes-.”

M: “Okay. Sorry I am jumping around a bit. So much to ask actually, and I want to make sure I come back-. What about the- the **movement** of the robot in the ward ah? I want your thoughts about the speed and whether it is a source of danger. Speed, what do you think speed wise?”

[Short pause. Mumblings and “Erm” heard in the background.]

M: “IDI11?”

IDI11: “So far okay la, from what I see. Because erm, when it face obstacles it will stop immediately also. So, so far is okay for speed.”

M: “I see. Other thoughts ah? Speed wise?”

IDI15: “We try to- what you call that ah, the back [IDI13: “Emergency stop.”]. Ya, **emergency stop** ah, actually the robot will jerk a bit. Ya, so I not sure if is really stable la. Because that time we just stop it… not so abrupt ah, [M: “I see.”] but yes the robot still tilt(s) a bit la.”

IDI13: “Another- [M: “Yes, please go ahead.”] another thing about movement let’s say robot really in my way right, then maybe need to rush. I- I want to like- I don’t know how to remove it to one side, you see. Can we control it? To where we want it to be. “

IDI12: “Because a bit heavy la [IDI13: “Ya.”] Have to be- ya.”

M: “Especially in the busy ward during the busy part [IDI13: “Correct, yes.”] of the day right [IDI12: “Yes, correct.”]. If you have to push the patient through the wheelchair [IDI13: “Ya.”], then there’s a medication trolley on the other side [IDI13: “Yes.”], so there’s no way to kind of gently move the RNA [IDI13: “Yes.”]. Uh-huh. Okay, that’s a good- important point.”

IDI14: “Same, I think also quite, erm I think let’s say in an environment whereby you have a lot of geriatric patient la, so taking into consideration, that **speed** is generally quite okay, ya. Because is quite slow ma. But can be a bit- the other aspect is that it can be too slow, sometimes if we want to have function of being able to deliver things, you want it to be maybe a bit faster, ya so that it can get the items to the patients at a faster speed la. Ya, so I think chicken and egg lorh, ya.”

M: “Um, do you think that based on the way they- the RNA adapt to the busy environment, has it been able to pause and start erm, in a safe way in general? Do you- or- and do you have other safety concerns ah, about the RNA because you have been seeing it in the ward 4- ward 4 or so a couple of times already. Any thoughts from the safety point of view? Other thoughts, I mean.”

IDI14: “Erm, I think at the moment because our ward is actually quite small, the space which is quite **narrow,** so there’s actually a lot of movements that we really need to navigate it la, because you are pushing off the bed, angle it so that the robot able to navigate through the pillar and the corner to the bed which is erm- which is blocked by the pillar. Ya, so then there’s some maneuver part that needs to be done la, ya.”

IDI13: “I would say if the robot is **too far** from the patient, is there a way for- for us to control it, ya because it’s really quite bulky to have to shift it.”

M: “To- to shift the bed or whatever it is to the patient-.”

IDI13: “To shift the robot.”

M: “To shift the robot closer to the patient right? [IDI13: “Ya.”] Alright, and is it able to navigate in a fluid way through either the table and- or maybe chair?”

IDI15: “Before they implemented they marked out right? Where the robot will go. That’s why what IDI14 say ah, we really need to move the bed a bit, provide the {box?} (12:55). Ya, so there’s a lot of movement for the robot to be able to navigate around, especially around the cubicle area.”

M: “So in a sense it’s a bit artificial right? Because we have to, we meaning the ward staff will have to make space for the RNA ah. [IDI13: “Ya.”] I see. Okay. Coming to the three functions ah, the vital signs seems to be, I mean IDI15 say that she thinks vital signs is the most erm…”

IDI15: “Out of the three tasks la.”

M: “Of the three is the most desirable thing. What about you all? What do you all think ah, when you observe the vital sign taking ah?”

IDI12: “**Vital sign** taking is so far… I think should be okay la, because you put the hand only {cleaning?} (13:35) purpose only. So far is no issue. But only thing that she asking for whether you got any pain or not, ya that is a bit- how the patient able to respond, how she capture that oh, patient will say ‘I no pain I got pain’. Ya, you ask that question ‘oh do you have any pain’, that’s good. But what if the patient respond ‘I got pain’, what is the consequence of him receiving the message from the patient. This is- is there any record that oh okay, patient say that ‘I got pain’, will say ‘what is your pain?’ or any conversation with the RNA and patient. [IDI13: “Follow-up questions.”] Ya, ya. So that is good that it asking ‘oh, do you have pain?’. Then let’s say patient say ‘I got pain.’ Then how would we- the robot will respond to this kind of question. [M: “Um.”] Ya, that’s our thinking la.”

IDI11: “And- and another one is erm, the robot will ask the patient erm… I am taking your **respiratory rate**-. [IDI12: “Ya, may not be-.”] Ya, because you can breathe faster right? If you telling me you are taking my RR, then I can breathe faster or breathe slower [IDI12: “Ya, ya, correct.”] So it shouldn’t be tell the patient that-.”

M: “That- there’s-. Makes them self-conscious la.”

IDI11 and IDI12: “Ya. Yes. Ya.”

M: “I see, I see. That’s a good point.” [IDI12: “Ya.”] Other thoughts ah, about the vital sign-.”

IDI13: “Will the robot like- **alert** the nurses? If there’s abnormal results, will like-.”

Note taker: “So like- sorry ah, so if let’s say the patient says erm, there’s a pain score of more than four then it will be alerted. So like there will be a- this alert on the dashboard la. So the nurses will be alerted.”

M: “Um, that’s going to be built in the future.”

Note taker: “Um. So for now actually it will also like show on the dashboard already, it would be highlighted as a red color wording. [IDI12: “Oh, for the pain.”] Ya.”

M: “Do you think the platform and the way the vital signs are being taken, is it- do you have any thoughts on that ah? Is it awkward, is it as you imagined it to be? Like because the robot opens up its chest and something comes out, is it scary? Like alien movie? [IDI1_participants laughed.] I- what are your thoughts ah?”

IDI15: “I think because our- the type of patient who are using RNA is those coherent la, so it’s okay. Ya, but if you… if you go and erm, implement this type of robot for those who got some mental problem of course cannot ah, they cannot follow and they might feel scared, ya. So it’s also important when we implement this type of robot you will share with the patient during orientation that we have this type of robot that take your vital sign, so this is what will happen, so they got an idea. Cannot be we just let the robot run away in the wards just like that (laugh). Ya, and I think with what she say ah, with regards to taking the **pain score**, I think now the robot can only recognize the scale of zero to ten right? So she’s right, when the patient say ‘I got no pain’, the robot won’t recognize it right? [M: “Um.”] Because most of the time you ask patient, you know they {say} ‘No, no pain.’ “

IDI12: “Ya.”

M: “Right, right that’s true. Other thoughts ah? About the platform and the apparatus ah? For vital sign monitoring?”

[Long pause]

IDI14: “Actually I find that it’s quite impressive already la, for a robot able to do your pulse rate and your saturation.”

IDI12: “I feel that patient and caregiver will love it la, because the robot is a new thing then everyone look and say ‘eh that’s a new thing’, it will be good that technology will be- make them to be happy as well, ya. (laugh)”

IDI13: “It’s quite- I think **entertaining** also like [IDI12: “Ya, I think carer will be like-.”] for the patients to be- lie down the whole day and just watching the robot- you know there’s some activities.”

[IDI1_participants laugh]

M: “I notice that the patient would wave ‘eh, come to me.’ [IDI1_participants laugh, IDI12: “Ya.”] That’s true. So the- the vital sign measurement part is not awkward, you mean patients can understand is it? The moment the platform comes out and all that, the probe and so on? Generally, patients are able to understand what they need to do ah?”

IDI12: “I think generally patient know la, we are- normally we take vital sign is the BP and the heart rate, ya. Usually they know temperature and BP and heart rate all that.”

IDI13: “The only limitation I think would be **language** la, because it’s-. [IDI12: “Ya, because it is in {inaudible word – 18:00} so not everyone will know English.”] They won’t really speak English.”

M: “I see, ya that’s true. Okay, so in general the design aspect as IDI14 said is quite impressive la, for taking pulse rate and heart rate, erm I mean er temperature-.”

IDI14: “Temperature-?”

IDI13: “Temperature there’s infrared.”

IDI12: “Ya, infrared. Ya, infrared ma, I saw.”

M: “What about the medication delivery aspect ah? IDI15 of course thinks it’s- really doesn’t add a lot of value, what do you all think ah?”

IDI14: “I think generally medication- because **medication** process is very- it’s rather complicated la. So the thing is about making sure that the medication is identified- given to the patients correctly that is one. Then the other thing it’s not just giving of medicine, it’s a lot about teaching and education, erm that is the other component la, about even the process of the nurse giving medication. Ya, so is not just the medicine then that’s it. Ya, and then the other thing is also ensure that medicine is actually being taken, being fed and being given safely, so then you’ll complete the process of administration process, ya. Not just the serving part of it la. It’s the consumption that’s important, ya.”

M: “IDI11, what do you think ah? The meds delivery part?”

IDI11: “Ya correct la, I agree with what IDI14 say, ya. We have to make sure the patient eat the medication also, then sometimes the patient will ask us what is the medication for, what is the indication ya, so you have to explain to them what is the meds for. So it’s not just to serve meds to patient.”

M: “IDI13, what do you think ah? Can- is it help- it sounds like at least the three colleagues here already say it’s not that helpful. Is-.”

IDI13: “Because it seems like it’s just delivering an- a normal general item. Ya, there’s no like value-add to **medication** serving component.”

M: “So if we stick with this medication delivery and think more broadly ah, what would you want to do to make the robot be better at helping with this task ah? And if you step back, is there anything the robot can actually do ah? Because there’s verification, there’s education and someone mentioned- I think IDI14 mentioned you have to observe the patient take the medication as well. [IDI14: “Yes.”] That means the robot has to do all three is that correct? Ideally?”

IDI15: “A lot of components la. As what she said, you also need to educate the patient, not only identifying the correct patient, giving the medication to the patient la, you also need to educate, make sure that they will swallow the medicine la. That they don’t throw it or- ya. I think the medication administration part is quite complex for a robot to do la. [M: “Quite complex ah?”] Still need one nursing-.”

IDI13: “Sorry, okay when you key in let’s say for example I serve Panadol right, and the screen will appear with that picture and what is it for and the side effects. Something different.”

M: “Assuming that person can read English la. [IDI13: “Assuming ya, ya.”] IDI12, you were going to say something? About the medication?”

IDI15: “Ya, for serving **medication** is- for- for robot right, I feel very complex. Because, erm… ya just Sister mentioned la, make sure that correct medication and need the person know why he taking for this one. And then communication and teaching and understanding of medication before you take la, so robot may not able to contribute a lot of things to the patient. So like they- {inaudible 21:42}

M: “I see. I will ask this another way, which is can you imagine if you were an engineer to think of a way- because medication delivery takes up fair amount of time right? In the ward. I mean especially in the morning [IDI1_participant: “Um.”] Erm, if you had a robot, what would the robot be doing to help out ah? If you were the engineer yourself. Is there anything you can think of?”

IDI12: “Okay, one thing right, if your robot is serving only medication, the nurse prepare and just give only, what is the role of robot need to do for serving medication. Is the same la for us what right, check the name, check the IC, do you have any drug allergy, make sure that this medication for what. So the purpose of robot using medication is only the nurse prepare put inside the robot, compartment and go there and give. Or-.”

IDI13: “Actually I think the main bulk of time is the **preparation** of medicine [IDI12: “Ya.”], like need checking and everything. Ya, not really the delivering, I think. [IDI12: “Um.”] Ya.”

M: “Based on my own observation, the- the morning round of medication delivery, it takes a fair amount of time right? Because you need at least one other nurse to double check right? Or is that not done anymore?”

IDI11: “Erm, it depends some of the medication la. So, ya, we have to check one by one medication if the medication need to countersign la. But if not, erm, one nurse can do it la.”

M: “I see. Switching to the so-called simplest task ah, delivering item like hot tea or snack ah, what do you all think about the robot’s role in this ah? What you’ve observed so far, any thoughts ah?”

IDI15: “Need to say. The nurse still need to prepare and put inside box right? [IDI13: “Ya.”, IDI1_participants laugh].”

IDI12: “The nurses will still need to prepare, put inside. So the robot’s duty is go there and send correct item.”

IDI15: “The robot can interact with the patient if the patient say ‘I want milo’, so the robot will recognize, go and make milo [IDI1_participants laughed.]. The nurses will still need to go and put inside the robot.”

M: “So the best thing is to provide a menu la [IDI13: “Uh, dispenser.”], milo, water, hot water, Coca Cola, like that is it? [IDI14: “Then it’s a water dispenser.”] The water dispenser, I see.”

IDI13: “Because again I think the main bulk of time is the preparation. Walking to the pantry and prepare it.”

IDI12: “Because the robot will want- patient will want ma, will take medication and communicate, taking this medicine then after that the robot go there, so the nurse prepare then you go there-.”

M: “But for hot drinks or snacks, isn’t there- isn’t it being prepared by a non-nurse person? No ah? Sometimes they’re just too busy is it?”

IDI15: “Only working hours la. After 6 like that ah, the nurse will do la. No more housekeeper on the ground.”

[IDI1_participants laughed.]

M: “Ah, anyway the housekeeper will be busy with other things during the day right?”

IDI15: “Ah, can be also. Ya, ya.”

IDI14: “Have to keep in mind that housekeeper has their own things to do. If the patient wanted something, we still have to attend to the patient immediately la. So that is when the nurses will just go and make the beverages that the patient actually requested.”

IDI15: “You must think how many patients you have in one ward ah, we only got one or two housekeeper.”

M: “So I want to divide the-the discussion into two- so the one is about the effectiveness of the task, one is whether it actually saves time. So if we just focus on the effectiveness, it seems like superficially, the measuring vital signs seems to be what the RNA does well enough to substitute what a nurse could do right? [IDI14: “If the-.”] The medication delivery and delivery of snacks and drinks is actually very hard to substitute, is that fair to say?”

IDI14: “Even- okay even in the vital sign monitoring, I think we also need to add erm, if we can, we can add in other components into it la, because it’s no point it can only do this one two three kind of vital signs monitoring whereas the other thing which is also important like blood pressure monitoring etcetera, it’s not in- it’s not combined so meaning the nurse will still have to do another set of vital sign monitoring, then it’s actually duplicating its- there’s no benefit to it la. So ultimately let’s say you want to do the vital sign, you can do the whole suite- the whole suite of it. Then you’re going to replace the nursing time of it.”

M: “Ya, in phase 2 they’re gonna do BP measuring and then- is it coming next week ah? [Notetaker]?”

Notetaker: “They said 10^th^.”

M: “Ya, okay.”

IDI14: “And then it definitely should not be like you go and put the BP cuff for the patient and you know those in ICare clinic they have to use that, that’s what I need to make sure.”

M: “Ya, well in the future there are technologies that measure blood pressure by looking at the retina, [IDI14: “Eye. Retina, eye.”] ya. But that one a bit science fiction still. Not quite here yet. Erm, what do the other thinks ah?”

IDI13: “Another thing about safety I think we talk about **hot water** right, let’s say we put a cup of hot water there and there’s a- maybe the robot needs to have emergency break. I think it will you know, cause some safety issues. Ya, because when it is moving, it seems a bit wobbly already. (laugh)”

M: “Okay, now in terms of the time saved ah, even… even-even though the- it takes time to prepare the hot snack- the snack or the hot drink, prepare the medication, vital sign probably less prep from you all, does it help to free up time for you all to do something else ah? Even though it might be doing very slowly, the robot. Does it materially… tentatively free up time for you all?”

[Long pause]

M: “That means it- so okay la, you have to prepare the Milo la, you put the Milo into the robot, then you don’t have to walk to the patient’s bedside. So that two- that 25 seconds- you can do something else-.”

IDI11: “But- but we still have to **add task** ma. We still have to add task, erm, have to- when you put into the- [IDI15: “Ya, program into the robot.”], yes, correct. So that time maybe, I can walk to the patient already.”

[IDI1_participants laughed]

M: “I see, what do the others-.”

IDI15: “We are not sure also if really ah, we want to implement it, who will do all these task assignment, is the nurse? One-by-one you go and register the patient also upon admission, you go and take photo, how many angle. Who- who will do all these things.”

M: “It’s a good question-.”

IDI14: “Ya, ya, ya, removing some time of the nurse to do certain tasks but this will replace by all these medication everything is- is just-.”

M: “I mean there are ways to streamline it. So let’s say if the robot really is going to be **mainstreamed**, then the moment the patients get admitted for admission at UCC, they already take photo and register and make sure the wrist tag and the photo is aligned. So when you just press RNA bed, cubicle 3 bed 2, that’s it la. But at the moment it’s done one-by-one la, which is a lot of effort right?”

[Long pause]

IDI14: “Which means you’re making one more **manpower** just to do that task la, of managing the dashboard ah?”

M: “But if- if the RNA is able to routinely collect vital signs, even though it does it very slowly, it- does it actually- will it actually help to free up time ah, for you all?”

IDI14: “You are asking about the ideal state ah?”

M: “Well even when it’s working very slowly la, like right now. [IDI15: “Can ah.”] How much time does it actually save ah? The vital sign bit. One minute, two minute per patient? Do you have any sense ah?”

IDI15: “It’s very straightforward, patient no questions or what, I think less than a minute can do one set for one patient ah.”

M: “One-minute ah?”

IDI15: “Ah. Everything like touch and wait for it to read.”

M: “I also notice that we, we meaning the nurses, we all- we actually multi-task right? [IDI1_participants agree in the background, laugh.] We put the thermometer in bed 1 and we already put up the-. Bed two, you know, we take down the thermometer in bed 1, then bed 2 we ask for pain score already. We multi-task all the time right?”

IDI1_unknown: “We’re very good at multi-tasking.”

IDI14: “It’ll be- maybe the robot can do everything by its own right, I mean if full function with robot doing vital signs, then he will just automatically go around the bed by itself, 1 2 3 4 5. Then finish already all the record will be recorded in the system, then move on to another cubicle, then go and do things by itself. Ya, then hopefully all this information can be **auto** transmitted la, to the documentation system. Ya, so then that will be more of a(n) ideal. And then nurses don’t even need to bother about doing the vital signs, but let’s say able to flag out things which is like [Unknown word 31:15, sounds like alarm] [IDI12: “Alarm (?), make it alarm ah, pew pew pew pew. That means that one abnormal. (laugh) Can what.”]. Ya, then that would definitely **save a lot of time**, meaning vital signs monitoring will not be part of the nurses’ work. Then it will just auto-flow by the robot.”

M: “Right now, forgive my ignorance, the patient vital signs are they measured all at the same time ah? In each cubicle?”

IDI12: “Yes, there’s standard timing.”

IDI12: “They got the timing one ah.”

M: “Or is it different patient different timing ah?”

IDI15: “No, there’s a standard timing, but ya. Some of the patients is more-.”

IDI13: “Depends on the acuity-.”

IDI12: “Depends on the acuity la.”

M: “The acuity right-.”

IDI14: “But generally you would have standard three rounds la, on average.”

IDI12: “Morning, 6 or 7am?”

IDI14: “Ya, 6, 11.”

IDI12: “6, 11, 6.30 early morning they start all vital sign have been taken. [IDI14: “One round.”] One round. Morning round.”

M: “Okay, so if it’s possible as IDI14 said that if they were to standardize 8pm cubicle 1, 8.10 cubicle 2 and you just take all 6 patients, then it may free up time for you all to do something else [IDI15: “the nurses la.”], it may-. How much time would that be ah? 5 minutes?”

IDI13: “15, 10?”

IDI14: “Quite a lot ah.”

IDI12: “Quite a lot ah.”

M: “10?”

IDI15: “Between there.”

IDI14: “Quite a lot, because you’re doing it every day leh.”

IDI12: “Every day, ya. Every morning.”

M: “Every shift I mean.”

IDI15: “Multiple times of a day.”

IDI12: “Especially in night shift ah, because early morning they are all- patients also wake up, the vital signs, so morning- early morning then it gets quite busy lor. Night shift- there is manpower also- 3 and 2 and 1 la in my ward. So morning 1am taking all the para, so at the point of time robot can help to take para okay la. Morning lorh.”

M: “What about the medication delivery? Since most of you are skeptical that it can really help, so is it correct to say there’s no point asking actually will save time for nurses? The- this-.”

IDI14: “Medication task-.”

M: “**Medication** delivery task. Wouldn’t save time is it correct?”

IDI14: “I don’t think so, because let’s say you want to deliver the medicine to the patient-. Okay, definitely there’s different type of patients that we’re talking about la. Then, erm we’re talking about the people- the person that can actually self-medicate so even if we deliver, the person can pour water and take- may not be a problem la. But we have- 80% of the population needs assistance in feeding medicine, ya so the robot will be just there with the medicine, but we’ll still need the pouring of water for the patient, feed the patient. [M: “patient might still be asleep.”] Correct, correct. The patient-.”

M: “Someone has to wake up the patient.”

IDI14: “Yes, and then complete the whole process of feeding the medicine for the- assist the patient to eat the medicine. [M: “I see.”] So even the act of pouring water la-.”

IDI13: “The act of taking the medicine out of the robot. One-by-one.”

IDI14: “Ah-ma must take- one-by-one. Feed them one-by-one.”

IDI13: “A lot of them are- a lot of them are also not so compliant la, not very excited to take medicine [Participants agree in the background], so may not even take it, so that will **cause more work** for us as well.”

M: “Um, okay. So what are- in the case of robot have all the medication inside him already, would it help ah? But the pharmacist will have to prepare la, is that correct?”

IDI15: “I think that one we need to look to the- talk to patient that can self-medicate, so that means they would be the ones to see the medicine on the robot’s screen, what medicine that I am taking. Ya, so we- it’s only catered to those coherent patients la.”

M: “Um-hm, and I assume that the third task, which is delivering hot drink and Milo and snack, that one, is it going to save any time at all?”

IDI14: “I think it will, but because the **compartment** is currently very small, so it’s a lot of limitation as to what things can be put inside, so many a times they may just want pajamas, baju, bedsheets and all that, ya which is quite frequent items that they may want. So if you can accommodate a **bigger** compartment so that that may be more helpful, ya.”

IDI15: “And when it comes to **beverage** ah, most of elderly they want **hot** one. For this cannot put cup right? It should be the top part [IDI14: “Plastic right?”] or bottom one, so ya, I think the elderly won’t like it la. They want hot drinks.”

M: “Um, I think IDI12 or IDI13 mentioned hygiene, is hygiene a concern ah?”

IDI13: “Yes it is. [IDI12: “Ya.”] Because usually for- after every patient, we would do a rub-down la. [M: “Right.”] For the BP cuff especially and everything else la, that is in contact with the patient.”

M: “Right, right. So that’s another area that could be a barrier to adoption la.”

IDI13: “Yeah.”

M: “Erm, in the final 10, 15 minutes, I would like to talk two other items, one is based on your observation ah, is- are patients generally responsive, scared, puzzled by the RNA ah? What are your observations like?”

[Short pause]

IDI11: “So far, erm… the patient like quite **excited** when they see robot . Ya, they like want to interact with it la. But then most of my patient are quite **elderly** also, so when you ask the question maybe, she’s not answering you or answering something that not related la. So when the patie- when the robot ask ‘do you have pain’ or something, then maybe the patient is not answering the- that question? Just talking some unnecessary or irrelevant one, so maybe need to put for it la. And if the patient is still sleeping like 5 o’ clock or 6 o’ clock, we taking the vital sign right, erm will it be erm- will the robot wake up the patient? [IDI14: “They will scared-.”] Ya, will **scare**- one-by-one. [Participants laughed in the background.] Ya, ya.”

M: “Suddenly the eye like that- like that.” (laugh)

IDI12: “The eye-.” (laugh)

M: “Okay, good point, good point. IDI12, what have you seen ah? Are patients generally okay with the robot?”

IDI12: “Because the- the- the robot testing the person he- I think he is age of 48 years old gentleman, so he was quite happy, very excited, ‘Oh ya, it’s very good. Robot is being helping to all of you.’. So he can move la, so for different patient la, because elderly may **not be suit** la huh. So this kind of age people able to take everything and then taking para, they really appreciate for the technology la. So this kind of group of people really, oh robot give the medicine also they will take. (laugh) So, ya.”

M: “IDI14 and IDI13, what do you think ah?”

IDI12: “They happy la, I see them.”

IDI14: “I-I- okay, I think for me when I see the robot, really- I also feel very excited to see something new in the institution, ‘eh, we are moving into something more **innovative** in our approach to care for our patients’. So I think for me myself I feel very- there’s a(n) element of excitement, I think definitely we also see that in our patients who are- I would say more like what IDI11 say, those with cognitive impairment may not understand or appreciate what is that thing you see. But for people who are able- who are younger, are not so unwell, they may be able to appreciate. They definitely see the appreciation la of a new technology that’s in the ward itself, ya.”

M: “I see, IDI13 do you- but do patients all understand the- the RNA functions or do you all have to additionally explain what it’s doing?”

IDI13: “I think we have to. Even if the patient understands English and is cognitively erm- [M: “Not impaired, um.”] impaired- not impaired, erm we still have to **orientate** the patient a little bit.”

M: “I see, I see. So it’s not erm, it’s still something that has to- will need some orientation la.”

IDI13: “Yes.”

IDI15: “Because it’s new. Because it’s new on the ground. So maybe if it’s running for quite some time, then… they will know [M: “That’s true, that’s true.”] what the robot is doing la.”

IDI14: “Ya, ya. I think like maybe let’s say in Changi Airport, if the robot roams around they will know that, ‘oh, this is more for information’, any FYI information, but if the robot roams in a hospital setting then may want to ask ‘eh, what is this for, what’s the purpose’.”

M: “My second to last question is what do you think about the **physical appearance** ah? And do you like it, you don’t like it, what would you like to change it? Is it too big, is it too short, is the voice too soft, you know? Anything, any thoughts ah? About-.”

IDI12: “Robot right- ya. Because you put the compartment right, it will close one, so we cannot see. Inside you can maybe put something can see inside, through one [M: “Transparent.”] Ya, transparent. Because it will close ah, but actually you can put the compartment because inside got small compartment only. You make it like, behind you say that can take the clothes or Baju right, there is no place to put ma huh. Ya, so transparent will be more nicer la. Maybe the robot hand everything you can put or some of the compartment that can create la. That is- (laugh).”

M: “IDI15 leh, because you started from the beginning. Do you think the design is like what you wanted?”

IDI15: “I think the design is okay, it’s just that maybe too… [IDI14: “Too **bulky**.”] [M: “Too bulky, okay.”] too bulky, ya. Because the whole body – (laugh). Ya.”

M: “So it will be nice to-.”

IDI15: “Yes, yes. To have some curve. [IDI1_participants laughed.] [IDI12: “I feel like the compartment, because-.”] No la, because…ya, it’s just straight one ah, ya.”

M: “I see, I see.”

IDI12: “Only-only you got compartment, actually I see the robot body right, you can put a bit of nice compartment, can design.”

IDI14: “It will be nice if can serve meals for us.”

IDI15: “Can serve- arms, then got arms-.”

IDI12: “Ya, ya. You know like the hand, both the hands come, make it like-.”

M: “It would be nice if you make it more human-like, is that what you’re trying to say?”

IDI13: “Human-like ah…”

IDI14: “More like- no, actually the eyes ah, we actually went through many rounds of picking how the eyes gonna move, how the eyes would blink, ya. [IDI1_participants laughed.] So generally it’s very pleasing la, ya. [M: “Uh-huh.”] The features itself, the interaction, the eyes, I think it’s very pleasing to look at, and the tone also, ya.”

IDI15: “The facial expression, I think-.”

IDI14: “Ya, ya.”

M: “IDI13 leh, your thoughts about the physical-.”

IDI13: “Erm, when I first saw the robot, I mean- I was a bit disappointed. Because it’s quite big, but the compartments are a bit **small** for the size. So… ya. The expectation is not… ya, there. Erm, maybe if we can have some hooks or what, you know, to hang clothes outside of the robot, it may be.”

IDI12: “Ya, so ya. At the hand or back, you can- you can put a lot ah. Ya, correct.”

IDI13: “More space saving.”

M: “I see.”

[IDI1_participants laughed.]

M: “IDI11, what do you think ah? The physical appearance of the robot. Can it- are you happy with it?”

IDI11: “It’s too bulky, it’s very big and ya, correct la, like what IDI13 say, ya the compartment is very small. So, ya, maybe want to add some place for it, so that the cup and all the jar can put inside.”

IDI12: “Maybe you can put ‘My name is Florence, I can help you-.’ So you put there, {Unknown phrase: 42:45 – 42:47} la so people can read la. Oh my name is who, my name is IDI12, I am going to do for you this thing so you just point la. So that the patient also see, or the- even though other people also see. Oh this robot is serving this kind of thing now.”

IDI14: I think maybe the other aspect is erm, [IDI12: “Can ah, that’s what the design-. (laugh)”] let’s say the design on the robot can pro- just a thought that came to mind, the robot can proactively ask [IDI12: “Ya.”] the patient: ‘oh, I am here to serve you, is there something that you want? On the screen got 1,2,3,4,5’, then the patient may choose ‘okay, can you please get me some hot drinks’, then she press number 5. Then it’s patient initiated to the robot, then the robot would- not sure what kind of enhancement la, but will able to get the item and go to patient directly et cetera. Oh I need to have one more blanket, then she press blanket, then the robot able to navigate. But currently our hospital system a lot of doors. Ya, so may not be able to enter into the door. [M: “Right. Ya, that’s true.”] Ya, that is one limitation la.”

M: “If you were to- now that you have experienced the robot, if you were to start from scratch ah, if you can design with the engineer, what- what **functions** would you like the robot to do ah? That you think would be beneficial ah?”

IDI14: “I think one thing is **companionship**. [M: “Companionship, okay.”] Ya, because we- a lot of people in the hospital they are more lonely, we don’t have the time to engage the family- the patients la. With some activities or et cetera, so companionship is good, so that those who are more lonely can have engagement, they are able to do that interaction games or whatever with the patients. [M: “Okay, that’s a good suggestion.”] For companionship with robots.”

IDI12: “Maybe robot can sing the song, just press the button, Chinese song, what song, so go to the aunty ah-ma bedside, the robot will sing the song for them. (laugh)”

M: “And now can sing Chinese New Year song, [IDI12: “Correct, correct what.”] then open up got ang-bao coming out right?”

IDI14: “Maybe able to add trans- Google translator. [IDI12: “Ya.”]

IDI15: “I think it will be better if the robot is really the smart type of robot that can really interact two-way **communication**.”

IDI12: “Ya.”

M: “Okay. Two-way communication, socializing interaction. Other thoughts ah? What else do you think, if you imagine the future like, what other nursing tasks might be- benefit from a robot to help ah, now that you seen it in action?”

IDI14: “I’ll add one la, maybe will be able to help the nurses in **lifting** and turning the patient.”

IDI13: “Ya. Was thinking of that also.”

IDI14: “I just need to bring my turning robot with me, then one nurse plus the robot can lift the patient, turn and will be able to assist la, ya.”

M: “That’s actually happening [IDI14: “In nursing tasks right, ya.”] in nursing homes. Ya, robot helping to bring a patient to take a shower or bath.”

IDI14: “Ya, transferring uh. Ya.”

M: “Yes, yes. Actual robot, not just exoskeleton. Other thoughts ah? IDI11, anything else you want the robot to do in the future?”

IDI11: “Maybe the robot can erm, like kind of like CCTV, like we are doing **monitoring** in the ward. So maybe the robot can do it, they can catch the patient, whether did they start to walk or climb out.”

IDI15: “Like can tell you ‘the patient is climbing up.’”

IDI12: “Okay, bed 20 patient climbing out, like that lor (laughs). It will stand there, ya.”

IDI14: “Then you go and block the person.”

IDI15: “Block the person”

[IDI1_participants laughed.]

IDI12: “Very good.”

M: “So erm, my final question ah, taking into account all the good and bad things of the robot, do you think there is a future for robot in the ward ah? Any- based on what you’ve seen so far? Looks-.”

IDI15: “Taking the- those wards ah- like this lor, like vital signs monitoring, doesn’t really need some human touch per say ah, because if you go and say like showering ah, I don’t know how the patient will perceive, like robot showering them. I- I am not sure, but for vital sign, because it’s like quite simple task, ya, so I think it’s still doable la. Acceptable la.”

M: “Um. IDI14 what you think, that overall you think there is a role for robot to help with the nursing task ah?”

IDI14: “I think there is la, ya, there’s definitely roles for robots to be-.”

M: “Even though the time saved doesn’t seem that significant la. Based on what you’ve seen so far, is that fair?”

IDI14: “Erm…”

IDI15: “I think there is la, really there is. Like what she said also earlier on, those- like for those turning or lifting patient, instead of two nurse going to patient to lift, can be one nurse and the robot lor. [M: “I see.”] Ya, so you save one time of the nurse lor. Ya.”

M: “Other- other thoughts ah, about the robot and ward-related work ah?”

IDI13: “I think the nurses need to be confident that if the robot can carry out what they do. Otherwise we would rather do it ourselves. The robot also has to be very **consistent**, ya, if sometimes it does and sometimes it doesn’t, we will end up doing it ourselves. I think.”

M: “Right, I see. Consistently taking blood pressure accurately, for example. [IDI13: “Yes, accurately.”] Um. Final thoughts ah? IDI12 or IDI11?”

IDI11: “Erm, ya correct la. And also if the robot can be like smaller can squeeze in… ya all the patients there, we no need to move all the beds and all the obstacles for it [IDI12: “Ya, ya.”] to have a way to-.”

IDI12: “That would be on the other ward la. So nurses when that- oh rather I will move and keep the-.”

IDI11: “Ya.”

IDI14: “I think should be robot adapt to the environment, not the environment adapt to the robot. [M: “Right, right.”] [Participants agree in the background] The people also.”

IDI13: “I think we also observe the slippers on the floor which was obstructing the robot. (laugh) So-.”

M: “Even something as small [IDI13: “Ya.”] as that can be an obstacle right?”

IDI13: “Ya, it’s extra work for us to have to move the slippers, because usually we don’t need to do that.”

M: “Right, that’s a good point. [Note taker], any other questions that I have missed ah?”

Note taker: “Erm… don’t think so.”

M: “That is quite a rich discussion.”

[Participants laughed in the background.]

IDI15: “On time, on time. On time.”

M: “Just few minutes before time.”

IDI15: “Ya, 4 minutes.”

M: “Thank you so much ah, I know everyone is busy. And then you’re getting off shift some more. 不好意思 (Sorry)! So erm, so the next phase is that they’re gonna move back the RNA and now there’s a BP cuff right?”

Note taker: “Erm, so it will be an oximeter la, so they’re just changing the oximeter to something that can measure the blood pressure.”

M: “BP ah, okay. So it’s still quite straight forward, just the finger and the- ya. [Note taker: “Yes, yes.”] And we’re going to continue in ward 4 right?”

Note taker: “Yes.”

M: “Are our nursing colleagues going to observe further, no hor? No more already?”

Note taker: “Erm, we will get other nurses to observe as well.”

M: “Okay, okay. That’s good. Thank you so much ah, thanks very much. And thanks very much for organizing and you know, help with the RNA.”
