# Supplementary material for: An Assessment of an Inpatient Robotic Nurse Assistant: A Mixed-Method Study
Source: J Med Syst. 2024 Oct 22;48(1):99. doi: 10.1007/s10916-024-02117-4 (PMC11496348; doi:10.1007/s10916-024-02117-4)
Supplement: Supplementary file 9 — Supplementary file9 (DOCX 44 KB) [file 10916_2024_2117_MOESM9_ESM.docx]

**PT001:**

| **Patient** | **Video number** | **Step** | **RNA Tasks** | **Timestamp**  **(mm:ss)** | **Description of Human-Robot Interaction (HRI)** | | | | **Coder** |
| --- | --- | --- | --- | --- | --- | --- | --- | --- | --- |
|  |  |  |  |  | **Verbal** | **Behavioral/Conduct** | **Facial Emotional Recognition (FER)** | **Visual (Attention)** |  |
| PT001 | VSM | 01 | Approaches patient and maneuvers to face patient |  |  |  | Smiled at the RNA when the RNA correctly maneuvers toward patient. | Patient kept his gaze on the RNA. | Cheryl |
|  |  |  |  |  |  | Sat upright with hands rested on his lap | Gave a slight smile upon RNA’s arrival | Maintained eye contact with RNA | YW |
|  |  | 02 | Greet patient, and its intention to help patient measure their vital signs |  |  |  |  | Gaze flickers between RNA’s video player and face – unsure which to focus on. | Cheryl |
|  |  |  |  |  |  | No physical contact/interaction with RNA | No sign of discrete facial expression | Eye contact shifted between video screen and face/camera | YW |
|  |  | 03 | Request patient to look at the camera (for facial detection and recognition) – Camera comes out. |  |  | body posture seemed stiff |  | Patient’s gaze shifted to look at the camera | Cheryl |
|  |  |  |  |  |  | No physical contact with RNA | No sign of discrete facial expression | Maintained eye contact with camera | YW |
|  |  | 04 | Informed patient of successful facial recognition. Camera shifts back |  |  | Fiddled with the wrist tag. |  | Patient’s gaze shifted back to the RNA’s video player | Cheryl |
|  |  |  |  |  |  | Fiddled with wrist tag seemingly in anticipation to scan | No sign of discrete facial expression | Gaze shifted to video player | YW |
|  |  | 05 | Request for patient to scan wrist tag |  |  | Was able to scan it at one go, smooth (before the RNA could give verbal instructions: “Please scan your wrist tag now”) – Patient could understand written instructions on the RNA video. |  |  | Cheryl |
|  |  |  |  |  |  | Was able to reach out and scan wrist tag comfortably | No sign of discrete facial expression | Eye contact shifted to bar code scanner | YW |
|  |  | 06 | Asked for patient’s pain score (“From 0 to 10, please tell me your pain score”) |  | Patient hesitated a while before saying “My pain score is 2” (seems like he wasn’t confident of doing it correctly.)  Repeated “My pain score is 2” with more confidence. | Stretched his hand to press on the screen (to indicate his pain score – might have thought it was touch screen).  Patient seemed confused for a while, and rather shocked. Nodded and did a hand gesture pointing from his mouth outwards – to show understanding to say out his pain score instead.  Finger pointed at the screen – seemed like he wanted to press on the screen to indicate pain score.  RNA did not capture pain score, prompted again. Patient nodded and repeated |  | Patient gazed at the RNA screen for a while  Patient gazed then shifted to the RA, when she informed patient to verbally say out pain score. | Cheryl |
|  |  |  |  |  |  | PT reached out to touch video screen, seemingly thinking it he was supposed to answer via touchscreen | Initially had a neutral expression, but seemed pleasantly surprised | Eye contact focused on video screen | YW |
|  |  | 07 | “The nurses would be informed of your pain score, I am glad that you are managing well.” |  |  |  | Patient’s expression remained neutral. |  | Cheryl |
|  |  |  |  |  |  | Gave a small nod | No sign of discrete facial expression | Glanced up momentarily, but otherwise his gaze remained fixed on the video screen | YW |
|  |  | 08 | Compartment opens, and request patient to insert finger into oximeter |  |  | Inserted his finger into the oximeter before RNA finished her verbal instructions.  Patient was unsure if he was doing it correctly. Patient spoke to seek confirmation. |  | Patient looked and read instructions on the RNA screened  Gaze flickered between RNA’s video screen and compartment when taking measurement | Cheryl |
|  |  |  |  |  |  | Reached out and inserted finger, but seemed unsure if he was doing it correctly. | Patient gave a small nod but otherwise his expression remained neutral. | Eye contact fixed on compartment | YW |
|  |  | 09 | Measurement completed, request patient to remove finger, compartment close. |  |  | Patient followed instructions smoothly. |  |  | Cheryl |
|  |  |  |  |  |  | Went smoothly, patient gave a small nod and retracted his finger | Expression remained neutral | Patient kept his gaze on the video screen | YW |
|  |  | 10 | Asking patient to keep still (for respiratory rate measurement) |  |  | Patient seemed to breathe with slightly more intensity than usual in the beginning. Was able to follow through instructions. |  | Patient’s gaze fixed on the screen. | Cheryl |
|  |  |  |  |  |  | PT sat still and breathed normally as per instructed | PT was pressing his lips together, but otherwise maintained a neutral facial expression | PT maintained eye contact with RNA video screen and occasionally flickered to RNA’s face | YW |
|  |  | 11 | Informed measurement completed, displayed vital sign measurement |  |  | Nodded to acknowledge the displayed VSM. |  | Patient gazed at screen | Cheryl |
|  |  |  |  |  |  | Read the results and gave a small nod | Expression seemed focused (on the results) | Maintained eye contact with the RNA’s video screen | YW |
|  |  | 12 | Informed patient of intent to leave, thanked patient |  |  | Patient’s head tilted to the left (towards the exit, upon seeing RNA’s screen indicating intent to leave).  Patient nodded, acknowledging RNA’s intent to leave |  |  | Cheryl |
|  |  |  |  |  |  | Gave a small nod and acknowledged that RNA was leaving | Relaxed facial expression | Maintained eye contact with RNA | YW |

| PT001 | ID/ | 01 | Approaches patient and maneuvers to face patient |  |  |  |  | Patient’s gaze follows the RNA as it moves closer towards the patient. Gaze flickers between the RNA’s video player and face – seemingly unsure which to focus on. | Cheryl |
| --- | --- | --- | --- | --- | --- | --- | --- | --- | --- |
|  |  |  |  |  |  | PT was sitting upright in his bed, with his hands rested on his legs | Neutral facial expression | Eye contact flickered between RNA’s face and video screen | YW |
|  |  | 02 | Greet patient, and its intention to deliver item to patient |  |  |  | Patient kept a neutral expression. | Gaze continues to flicker between the RNA’s video player and face – unsure which to focus on. | Cheryl |
|  |  |  |  |  |  | No physical contact/interaction with RNA | Neutral facial expression | Eye contact flickered between RNA’s face and video screen | YW |
|  |  | 03 | Compartment opens, request the patient to retrieve the item (“Please take your item from the compartment.”) |  |  | Seemed confident and was able to retrieve item smoothly, before RNA finished her instructions. |  | Patient looks at the compartment | Cheryl |
|  |  |  |  |  |  | Smoothly retrieved items before instructions were completed | Neutral expression | Maintained eye contact with compartment | YW |
|  |  | 04 | Compartment closes |  |  | Patient places the retrieved item at the bedside table, and faces the RNA. |  |  | Cheryl |
|  |  |  |  |  |  | Placed the item on the bedside table | Neutral facial expression | Eye contact switched between the bedside table and back to the RNA | YW |
|  |  | 05 | Informed patient of the successful delivery and its intent to leave, thanked patient. |  |  | Looked and fiddled with wrist tag. | Patient smiled when RNA thanked him. Nodded – acknowledged the RNA’s intention to leave. | Gazed at both the video player and closing compartment.  Gaze flickered between the RNA’s video player and face – unsure which to focus on when RNA informed intent to leave. | Cheryl |
|  |  |  |  |  |  | PT was fiddling with wrist tag | PT gave a small smile and noddle to acknowledge that RNA was leaving | Eye contact flickered between video screen and RNA’s face | YW |
| PT001 | MD/ | 01 | Approaches patient and maneuvers to face patient |  |  |  |  | Patient was looking at his phone | Cheryl |
|  |  |  |  |  |  | Patient sat upright in bed | Expression remained neutral | Eye contact was on his phone | YW |
|  |  | 02 | Greet patient, and its intention to deliver medication to patient |  |  | Patient looked up at RNA for a while and looked back at phone briefly (rather hesitantly) to press the camera function on phone, passed phone to RA (to take photo) – seemingly impressed by the RNA |  |  | Cheryl |
|  |  |  |  |  |  | Patient was fiddling with phone and turned on the camera function before passing it to the RA to take a photo | Seemed slightly surprised when he looked up at the RNA | Looked up from phone to RNA | YW |
|  |  | 03 | Request patient to look at the camera (for facial detection and recognition) |  |  |  | Patient smiled at the RNA | Kept his gaze steady at the RNA’s camera. | Cheryl |
|  |  |  |  |  |  | No physical contact/interaction with RNA | Expression remained neutral | Kept his gaze on the RNA’s camera | YW |
|  |  | 04 | Informed patient of successful facial recognition |  |  |  | Neutral expression. |  | Cheryl |
|  |  |  |  |  |  | No physical contact/interaction with RNA | Expression was relaxed | Eye contact was on video screen | YW |
|  |  | 05 | Request patient to scan wrist tag |  |  | Patient was fumbling about for a while to find his wrist tag on his bed.  Was able to scan it at one go, smooth (before the RNA could give verbal instructions: “Please scan your wrist tag now”) – Patient could understand written instructions on the RNA video. |  |  | Cheryl |
|  |  |  |  |  |  | Patient was looking around his bed and subsequently the bedside table, looking for his wrist tag.  Once he found it, he was able to scan it smoothly. | Expression remained neutral | Eye contact was around his bed and bedside table while looking for wrist tag, before flicking back to the RNA’s scanner | YW |
|  |  | 06 | Compartment opens for patient to retrieve medication. |  |  | Patient’s gaze flickers about the compartment and the RNA’s video player for a while. Was intuitive to patient to remove the medication from compartment as patient was able to do it smoothly before the RNA could provide verbal instructions (“Please take your medication from the compartment.”)  Had to bend his body and stretch out hand a bit to retrieve the medication. |  |  | Cheryl |
|  |  |  |  |  |  | PT had to stretch his hand out, but was able to confidently remove medication before instructions completed and placed it on bedside table. | Expression remained neutral | Eye contact switched between bedside table and compartment | YW |
|  |  | 07 | Compartment door closes, request patient to consume medication |  |  |  | Patient kept a neutral expression. | Gazed at the RNA’s video player. | Cheryl |
|  |  |  |  |  |  | No physical contact/interaction with RNA | Expression remained neutral | Eye contact on RNA’s video screen | YW |
|  |  | 08 | Informed patient of the successful delivery and its intent to leave, thanked patient |  |  |  |  | Gaze remained fixed on the RNA’S video player, until patient retrieved back phone from RA (who has taken video of his interaction with the RNA.) | Cheryl |
|  |  |  |  |  |  | Placed his hand out to retrieve his phone from the RA who was helping him to take photos. | Expression remained neutral | Eye contact switched from the RNA’s video screen to his phone. | YW |
